# CTCF loss mediates unique DNA hypermethylation landscapes in human cancers

**DOI:** 10.1186/s13148-020-00869-7

**Published:** 2020-06-05

**Authors:** Nathan A. Damaschke, Joseph Gawdzik, Mele Avilla, Bing Yang, John Svaren, Avtar Roopra, Jian-Hua Luo, Yan P. Yu, Sunduz Keles, David F. Jarrard

**Affiliations:** 1 Department of Urology, University of Wisconsin School of Medicine and Public Health, Madison, WI USA; 2 Waisman Center and Department of Comparative Biosciences, University of Wisconsin, Madison, WI USA; 3 Department of Neuroscience, University of Wisconsin, Madison, WI USA; 4 Department of Pathology, University of Pittsburgh School of Medicine, Pittsburgh, PA USA; 5 Department of Biostatistic and Medical Informatics, University of Wisconsin, Madison, WI USA; 6 University of Wisconsin Carbone Comprehensive Cancer Center, Madison, WI USA; 7 Environmental and Molecular Toxicology, University of Wisconsin, Madison, WI USA; 8 7037 Wisconsin Institute for Medical Research, 1111 Highland Avenue, Madison, WI 53705 USA

**Keywords:** CTCF, DNA methylation, Cancer

## Abstract

**Background:**

The chromatin insulator CCCTC-binding factor (CTCF) displays tissue-specific DNA binding sites that regulate transcription and chromatin organization. Despite evidence linking CTCF to the protection of epigenetic states through barrier insulation, the impact of CTCF loss on genome-wide DNA methylation sites in human cancer remains undefined.

**Results:**

Here, we demonstrate that prostate and breast cancers within The Cancer Genome Atlas (TCGA) exhibit frequent copy number loss of CTCF and that this loss is associated with increased DNA methylation events that occur preferentially at CTCF binding sites. CTCF sites differ among tumor types and result in tissue-specific methylation patterns with little overlap between breast and prostate cancers. DNA methylation and transcriptome profiling in vitro establish that forced downregulation of CTCF leads to spatially distinct DNA hypermethylation surrounding CTCF binding sites, loss of CTCF binding, and decreased gene expression that is also seen in human tumors. DNA methylation inhibition reverses loss of expression at these CTCF-regulated genes.

**Conclusion:**

These findings establish CTCF loss as a major mediator in directing localized DNA hypermethylation events in a tissue-specific fashion and further support its role as a driver of the cancer phenotype.

## Background

Primary prostate cancer (PC) is a common age-related disease with low overall mutation rates compared to other cancers [1]. Prostate tumors exhibit epigenetic instability marked by frequent hypermethylation at CpG islands and global DNA hypomethylation [2]. Gene silencing precedes DNA hypermethylation, where evacuation of activating transcription factors is associated with alterations to chromatin structure [2, 3]. Disruptions in chromatin organization are a hallmark of cancer. Understanding the drivers associated with nuclear organization, DNA methylation, and gene expression are key to our understanding of disease including the important question of why DNA patterns vary between cancers of different tissues.

CCCTC-binding factor (CTCF), a zinc finger DNA-binding protein, is largely responsible for bridging this gap between expression and chromatin organization. CTCF functions in gene transcription and repression and as an insulator that interferes with enhancer-promoter interactions [4, 5]. CTCF recruits cohesin to assist in these chromatin organization functions, including in long-range interactions between genes [6]. Between 55,000 and 65,000 consensus, CTCF binding sites are found in the human genome [7], of which ~ 5000 are highly conserved between species and tissues [8, 9]. Of the remaining CTCF sites, 30–60% demonstrate unique tissue-specific DNA binding patterns. CTCF binds to these target DNA sequences in a DNA methylation-dependent manner and functions as a boundary element important in maintaining methylation at specific sites [10–14].

These findings provide a backdrop for a putative role for CTCF in the genome-wide regulation of DNA methylation. Forced downregulation of CTCF expression results in altered methylation patterns at several tumor suppressors and oncogenes [15, 16]. In the prostate, the *Igf2-H19* locus experiences DNA hypermethylation with CTCF downregulation at a series of intergenic CTCF sites resulting in imprinting loss during aging and cancer development [17, 18]. CTCF is located on chromosome 16q22.1, a region of common deletion in many epithelial cancers including prostate and breast [19]. Using large-scale genome analysis technologies, we report novel insights into the role of CTCF functional loss in directing DNA hypermethylation in multiple human cancer types. We propose that CTCF loss provides a unique contribution to the cell-type specific chromatin landscape in cancer.

## Results

### CTCF knockdown in prostate cancer cells leads to hypermethylation at CTCF binding sites

**Fig. 1:**
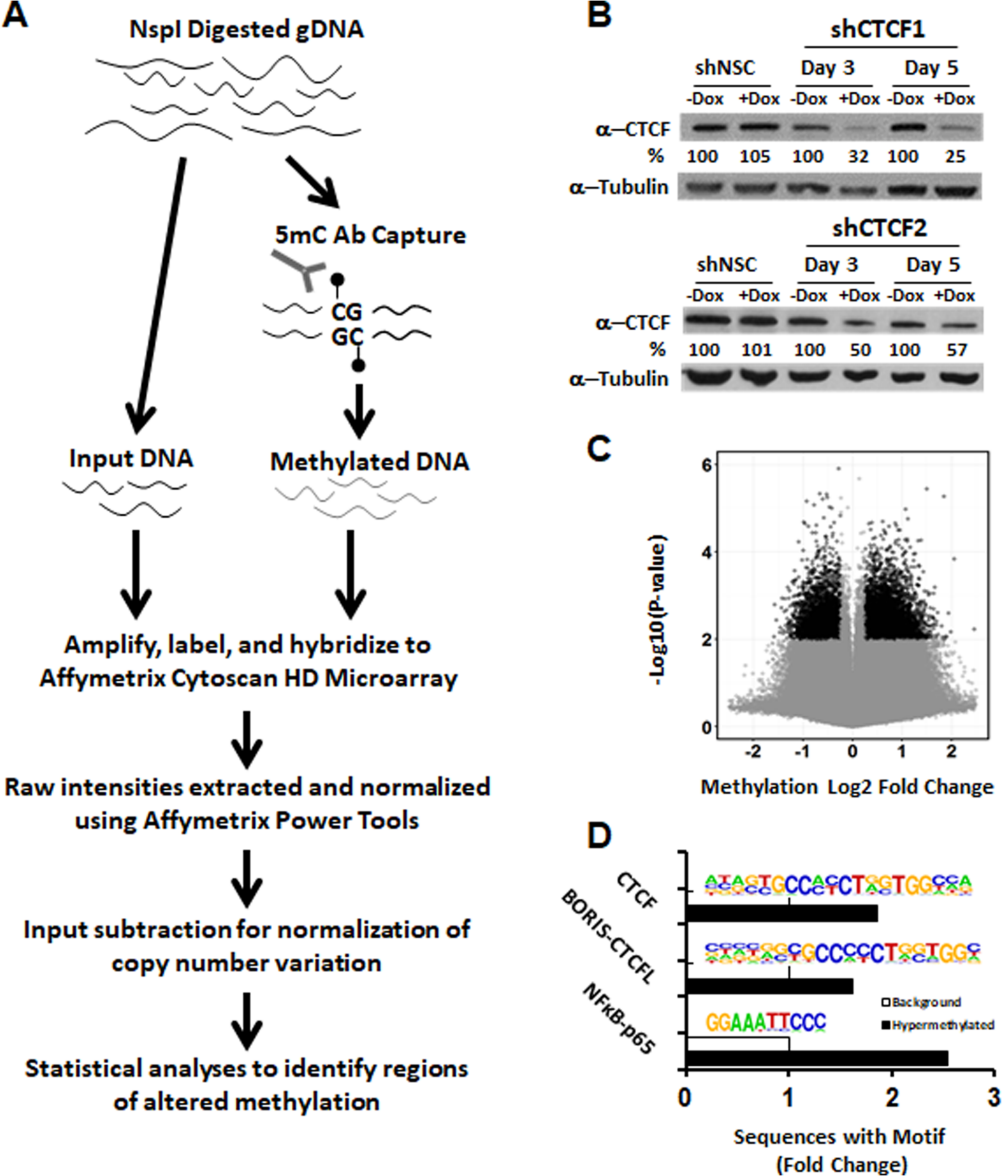
Knockdown of CTCF protein results in DNA hypermethylation preferentially at CTCF sites. **a** Workflow of methylated DNA immunoprecipitation followed by copy number array application (MeDIP-chip) for detecting methylation alterations. NspI restriction fragments were bound to anti-5-methylcytosine antibody, eluted, and hybridized to a Affymetrix Cytoscan HD probe. An unenriched total input fraction was processed for comparison. **b** Short hairpin mediated CTCF knockdown in two separate shRNA targeting CTCF verified by western blotting after 3 and 5 days of shRNA induction including shRNA non-silencing control (shNSC). Data shown are one representative of 3 independent experiments using immortalized HPECs. Percentage knockdown compared to shCTCF -Dox control, quantified by ImageJ. **c** Volcano plot of detected methylation changes in CTCF knockdown HPECE6/E7 after 5 days of dox exposure (cut-point, methylation Abs. Log2FC > 1.5, *P* < 0.01). **d** De novo motif analysis results using HOMER. Fold change enrichment of hypermethylated sequences was compared to array background. The top 3 transcription factor motifs included CTCF, BORIS (a CTCF paralogue), and NFκB-p65 (all *P* < 0.001)

We sought to determine whether decreased CTCF expression is causal in directing methylation to specific regions using a genome-wide methylation analysis that employs immunoprecipitation of methylated DNA followed by application to copy number variation arrays (MeDIP-chip) [20, 21] (flowchart Fig. 1a). We employed the CytoScan HD copy number variation array with balanced whole-genome coverage and focused on probes with CpG density > 2.5% and methods as previously described [20, 21]. Microarray raw data and processed data has been deposited on GEO, accession number GSE93328. To knockdown CTCF expression, we used a doxycycline-inducible lentiviral vector containing either one of two separate short hairpin-mediated RNAs (shRNA) or a non-silencing control. These vectors were stably integrated into the immortalized, non-tumorigenic PC cell line HPECE6/E7 that robustly expresses CTCF and is non-tumorigenic [22]. Decreased protein expression was verified by western blotting after shRNA induction (Fig. 1b). Methylation of six differentially methylated probes from the array using both MeDIP-qPCR and COBRA techniques validated the results of the MeDIP-chip (Additional file 1: Fig. S1A-F)

Methylation profiling yielded 9640 differentially methylated regions (DMRs) between CTCF knockdown and controls (*P* < 0.01; Absolute FC > + 1.5). The DMRs in the CTCF knockdown genome demonstrate 60.2% of these probes develop new hypermethylation (Fig. 1c). Across all probes, there was an increase in the Abs Log2FC magnitude of hypermethylation (0.63 mean absolute value) in the CTCF knockdown samples versus controls (0.55; *P =* 3.5E−37). Differentially methylated probes were then registered with respect to gene features using data provided with the array. Genome-wide distribution of DMRs across gene features including exons, introns, and promoters mirrored the total DMR distribution and favored hypermethylation, but no enrichment of any specific gene feature is noted.

We questioned whether the DMRs of CTCF knockdown cells had significant associations with CTCF or other transcription factor binding motifs. We performed a de novo motif finding approach of known transcription factor binding sites, using HOMER motif analysis on the DNA sequence of *NspI* fragments detected by the array probes as described [23]. Enrichment of CTCF, of BORIS/CTCFL (a CTCF paralogue), and of NFκB-p65 were the top sites found in hypermethylated sequences compared to the background distribution of the array (All *P* < 0.001) (Fig. 1d). Taken together, these data reveal CTCF loss of expression induces hypermethylation within CpG enriched regions, and this occurs preferentially at CTCF binding sites.

### CTCF knockdown preferentially alters genes that contain promoter CTCF binding sites

**Fig. 2:**
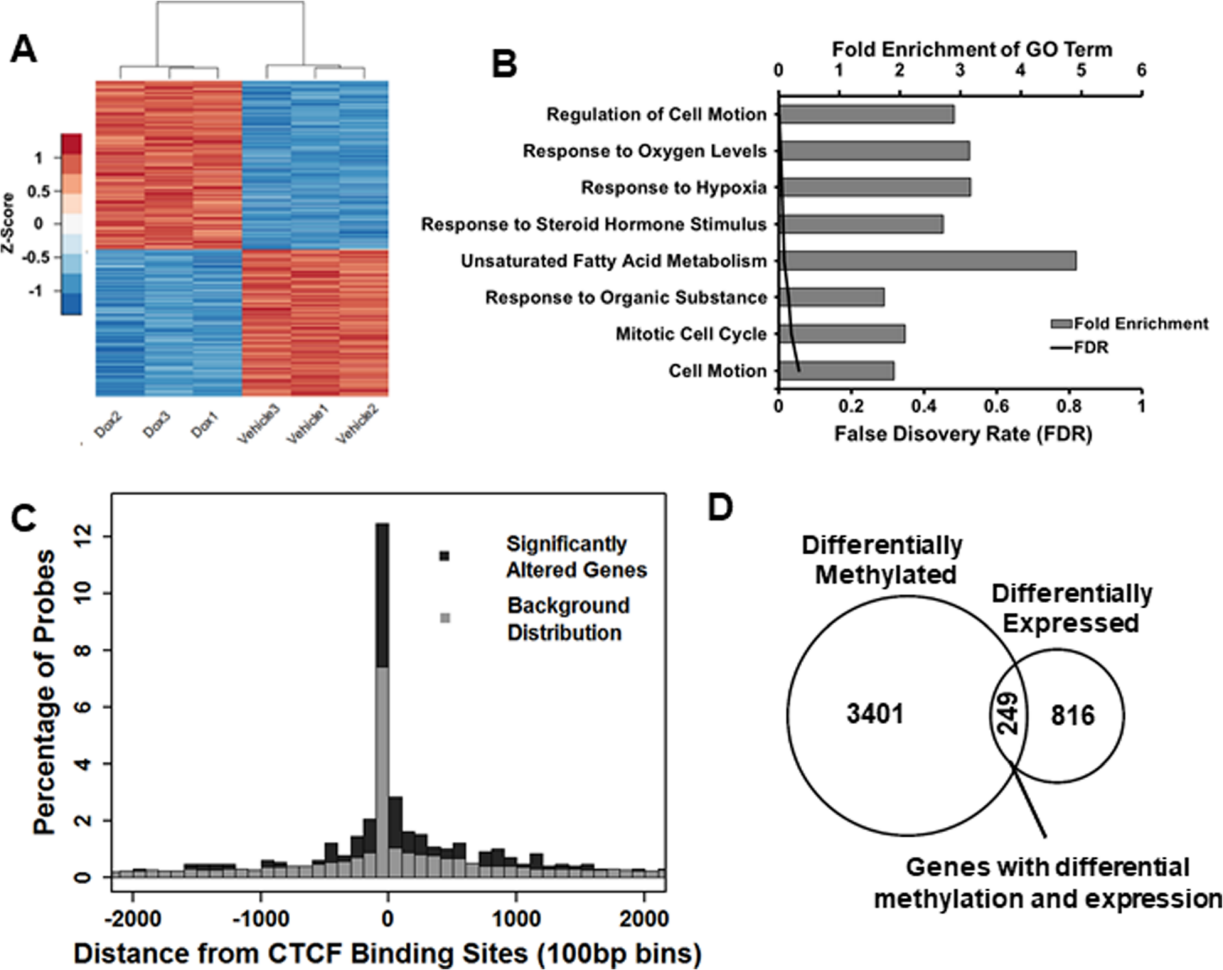
Transcriptional profiling of genes altered with CTCF knockdown. **a** Heat map of differentially expressed (DE) transcripts following 5 days of CTCF shRNA induction (Dox) versus uninduced vehicle control (vehicle). CTCF knockdown in HPECE6/E7 leads to 1308 significantly altered gene transcripts (FDR < 0.1). **b** Gene ontology (GO) analysis of DE genes, pathways with FDR *q*-value < 0.05. **c** Prostate cell CTCF binding sites (LNCaP ChIP-Seq) are enriched near transcription start sites (TSS) of DE genes identified after CTCF knockdown (*P* = 0.0001, Chi-square test for + 2 kb from TSS). **d** Venn diagram displaying overlap between differentially methylated genes and differentially expressed genes identified by arrays. Detected DMRs found within a promoter or transcribed region represented 3650 genes. Compared with 865 genes (865 genes from 1308 transcripts with gene annotation data), 249 genes were differentially expressed and contained a DMR (*P* = 1.1e−5; hypergeometric test)

Given the changes in DNA methylation at CTCF binding sites, we sought to determine whether CTCF loss alters specific genes and whether these genes contain CTCF binding motifs. We profiled CTCF knockdown and control HPECE6/E7 cells using Affymetrix Human Transcriptome 2.0 arrays. Microarray raw data and processed data has been deposited on GEO, accession number GSE93363. Compared to control, 1308 gene level transcripts (3% of total) were significantly altered (FDR *q* < 0.1) with CTCF knockdown corresponding to 608 downregulated and 700 upregulated transcripts (46.5% and 53.5%, respectively) (Fig. 2a). Validation of nine altered genes was performed using PCR to validate the array results (Additional file 1: Fig. S2A-B). We applied gene ontology (GO) to analyze phenotypic associations with all genes significantly altered with CTCF knockdown. Gene ontology analysis using both up and down differentially expressed genes indicated an enrichment of genes associated with cell motion, oxygen levels, and/or hypoxia and response to hormone stimulus (Fig. 2b) features important in cancer progression.

Publicly available CTCF ChIP-Seq data for the LNCaP prostate cancer cell line was used to define putative CTCF binding sites (GEO: GSE33213) near (+ 2 kb) the transcription start site (TSS) of genes. Comparing the genes, differentially expressed on our CTCF knockdown array and overlapping these with the CTCF TSS binding sites reveals an enrichment of altered genes around CTCF TSS sites (39%) compared to only 24% of genes otherwise covered by the array (Chi-square *P* < 1e−4) (Fig. 2c). This increase in significantly altered genes around CTCF TSS binding sites validates the function of CTCF as a locally acting transcription factor.

We next examined the overlap of differentially expressed genes and differential methylation marks. The 9640 DMR MeDIP-chip probe fragments were mapped to the 33,801 unique annotated RefSeq coding transcripts profiled by the transcriptional array. Of our DMRs, 61% (5877/9640) are found within transcription or promoter-associated regions (< 2 kb downstream and < 5 kb upstream). These DMRs together represent 3650 unique genes that we overlapped with 1065 differentially expressed annotated genes found on the expression arrays (243 genes lack annotation). Without respect to transcriptional direction, 147 genes had decreased expression (with increased methylation), and 102 genes had increased expression (with decreased methylation) all found to be associated with CTCF expression loss (*P* < 0.0001; hypergeometric distribution) (Fig. 2d; Additional file 2: Table S1).

### DNA hypermethylation occurs at CTCF binding sites within downregulated genes after CTCF knockdown

**Fig. 3:**
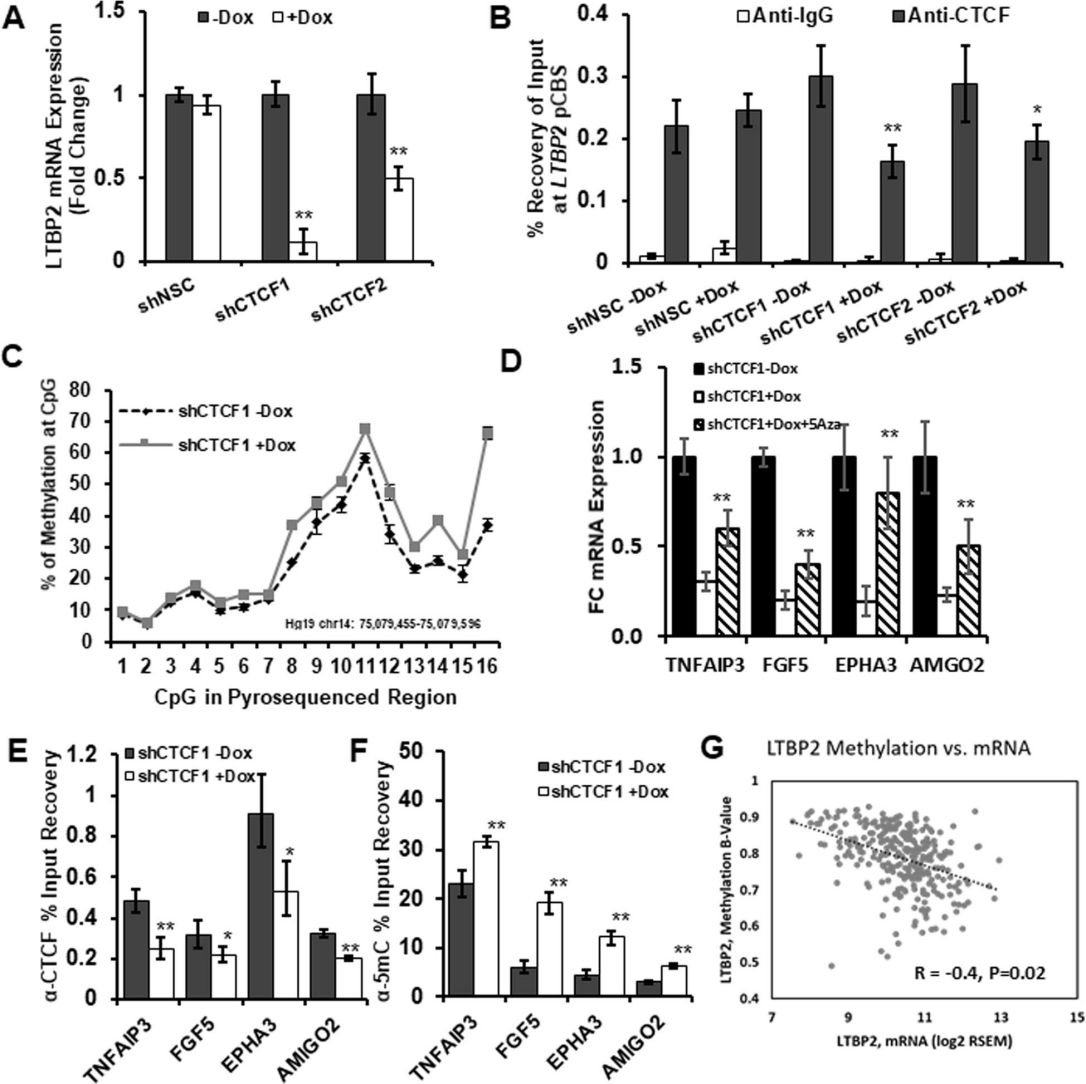
DNA methylation alterations occur at CTCF binding sites after CTCF knockdown in vitro and methylation inhibition reintroduces gene expression. Stable E6/E7 cell lines expressing CTCF shRNAs were cultured up to 10 days. **a** Validation of LTBP2 transcriptional silencing after 10 days of shCTCF induction by qPCR. Data are shown mean ± SD of technical triplicates from one representative experiment of three. **b** ChIP-qPCR for CTCF at LTBP2 promoter associated CTCF binding site (pCBS) ~ 400 bp upstream of LTBP2 transcription start site. Showing a reduction in CTCF binding after 10 days of shCTCF induction. Data shown are mean + SD of technical triplicates from one representative experiment of three. *P < 0.05 and **P < 0.01. **c** Pyrosequencing of bisulfite DNA demonstrating increased methylation at LTBP2 promoter CTCF binding site after 10 days of shCTCF1 induction. Data shown are mean ± SD of technical triplicates from one representative experiment of three. shCTCF2 induction and controls are shown in Supp Fig S4. **d** Decreased TNFAIP3, FGF5, EPHA3, and AMIGO2 transcriptional silencing after 10 days of CTCF knockdown. At day 5, Dox + cells were also exposed to 5-aza-2 deoxycytidine a methyltransferase inhibitor at a low 0.2 uM dose that does not result in significant growth inhibition. Data shown are mean ± SD of technical triplicates from one representative experiment of three. *P < 0.05 and **P < 0.01. **e** ChIP-qPCR for CTCF demonstrating decreased binding activity at promoter associated CTCF binding sites of candidate genes after 10 days of CTCF knockdown (for controls and expanded results see Supp Fig S3). **f** MeDIP-qPCR of promoter associated CTCF binding sites exhibiting loss of CTCF binding. Methylation increases were detected accompanying reduced CTCF binding. **g** Methylation *B*-values and mRNA (log2 RSEM) expression levels compared for LTBP2 gene in TCGA prostate tumors (Cell 2015). Pearson correlation *R*-value shown. Data was downloaded from cBioPortal for PRAD TCGA samples (Cell 2015). Decreased mRNA expression correlates with greater LTBP2 methylation

To further define the role of DNA methylation at these CTCF regulated genes, we examined whether CTCF knockdown would lead to promoter-CTCF binding site associated gains of DNA methylation after transcriptional silencing of target genes. From our list, we examined genes based on three criteria including greatest extent of expression downregulation, the presence of a conserved CTCF binding site within the promoter (LNCaP ChIP-Seq and ENCODE CTCF binding sites: UCSC Genome Browser), and a CpG-dinucleotide percentage of ≥ 2.5% within the identified CTCF site. These criteria identified five initial genes to be screened including *LTBP2* (latency TGF-β binding protein 2), a gene previously found to undergo promoter hypermethylation in cancer [24]. After verifying *LTBP2* transcriptional silencing using qPCR following CTCF knockdown at 10 days (Fig. 3a), we characterized CTCF binding at the *LTBP2* promoter CpG binding site by CTCF-CHiP. A significant reduction in CTCF binding to the *LTBP2* promoter in knockdown cells is demonstrated compared to controls (Fig. 3b), for other genes (Additional file 1: Fig. S3A-H). DNA methylation across this CTCF *LTBP2* region was then examined in control and CTCF deficient cells. Quantitative pyrosequencing of bisulfite DNA demonstrates consistent increases in DNA methylation across multiple CpGs within this promoter CTCF binding site (Fig. 3c and Additional file 1: Fig. S4A) but no differences with NSC controls (Additional file 1: Fig. S4B). We validated these differences using MeDIP-qPCR across this region which demonstrates 20–28% increase in methylation with both CTCF knockdown shRNAs (Additional file 1: Fig. S4C).

We then applied this analysis to other genes transcriptionally silenced following CTCF knockdown that met the above criteria. *TNFAIP3*, *FGF5*, *EPHA3*, and *AMIGO2* all demonstrated significantly decreased expression up to 10 days of CTCF knockdown (Fig. 3d). These genes develop decreased CTCF binding activity at the identified promoter CpG binding sites assessed by CHiP (Fig. 3e). MeDIP-qPCR demonstrates significant increases across these regions of decreased CTCF binding for all genes (Fig. 3f). These data indicate that loss of CTCF binding activity results in DNA methylation gains at specific CTCF binding sites within transcriptionally silenced genes.

To further determine the role of DNA methylation in this silencing of expression, treatment of the CTCF knockdown cell lines with low dose 5-deoxyazacytidine was performed. Utilizing 0.2 uM dose leads to inhibition in methylation without significantly altering proliferation. Exposure of CTCF knockdown cells (+Dox) to this demethylating drug beginning day 5 results in a reexpression of these hypermethylated genes when assessed at day 10 (Fig. 3d). Therefore, DNA inhibition is important in the underlying expression of these genes.

Finally, we examined whether DNA methylation within these CTCF promoter regions alters expression of these CTCF-associated genes in human prostate cancer. We queried 333 PC samples within the publicly available The Cancer Genome Atlas (TCGA) dataset. The Illumina Infinium HumanMethylation450 (HM450) data and RNA expression data for these TCGA prostate specimens were examined via cBioPortal for these five genes [25]. An inverse correlation between increasing DNA methylation and decreased mRNA expression is seen for *LTBP2* (Fig. 3g)*,* as well as*TNFAIP3* and *AMIG02* (Additional file 1: Fig. S5A-E). These experiments indicate a direct link between hypermethylation within genes containing a promoter CTCF binding site and a downregulation of CTCF expression.

### In vivo prostate and breast cancers CTCF copy number deficient tumors demonstrate increased DNA hypermethylation events

**Fig. 4:**
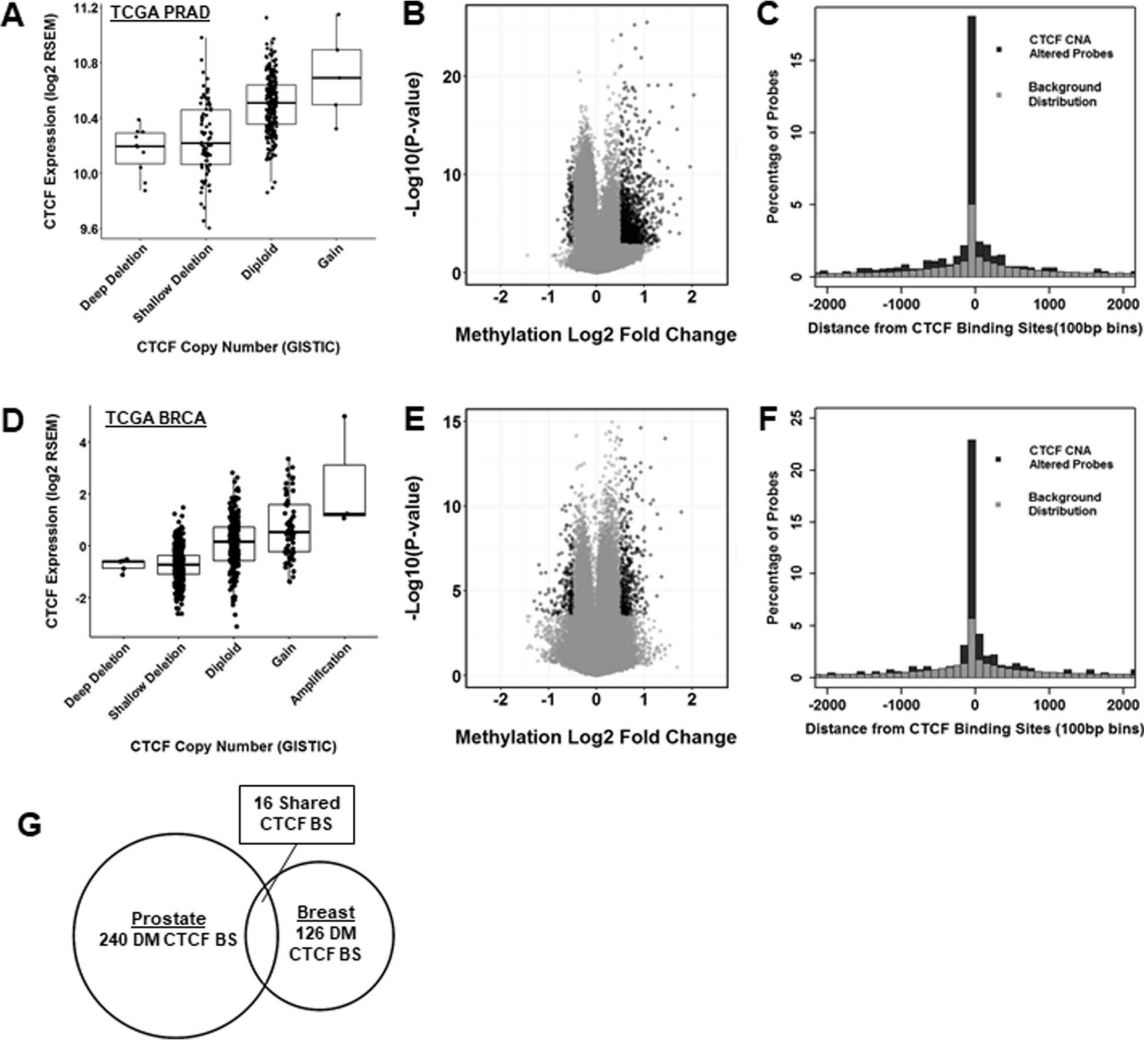
Prostate and breast tumors of the TCGA harboring CTCF copy number loss demonstrate hypermethylation events. Alterations exhibit a distinct DNA methylation profile. **a** Primary prostate tumors from TCGA (*n* = 333) segregated by CTCF CN status, boxplots of RNA-Seq for CTCF mRNA demonstrating significantly altered expression in diploid versus deletion cancers (*P* < 0.03). **b** Volcano plot of Illumina Methylation 450k Array data for CTCF CN loss tumors versus CTCF diploid tumors reveals increased primarily hypermethylation events, prostate tumors. Dots represent individual probes; Black, above cut point (Absolute value log2-FC loss/diploid *B*-values > 0.5, Adj *P* < 0.01). **c** PCa cell line LNCaP CTCF ChIP-Seq (GSE33213) identified putative CTCF binding sites. The black bar is percentage of differentially methylated probes, and gray bar is percentage of total probes from HM450 array were calculated with respect to proximity to CTCF binding sites. **d** Primary breast tumors from the TCGA (*n* = 816) segregated by CTCF CN status, boxplots of RNA-Seq for CTCF mRNA. **e** Volcano plot of 450k Array for BRCA tumors; black, above cut point (absolute value log2-FC loss/diploid *B*-values > 0.5, Adj *P* < 0.01). **f** BCa cell line MCF7 CTCF ChIP-Seq (GSE30263) identified putative CTCF binding sites for BRCA samples. The black bar is percentage of differentially methylated probes, and gray bar is percentage of total probes from HM450 array were calculated with respect to proximity to CTCF binding sites. **g** Overlap comparisons of differentially methylated (DM) CTCF binding sites in prostate and breast tumors demonstrate distinct methylation profiles at CTCF sites, related to Additional file 2: Table S2

We then questioned whether CTCF loss in vivo has an impact on hypermethylation patterns in human cancers. Level 3 RNA-sequencing, GISTIC2 copy number alterations, and HM450 methylation data were downloaded for 333 primary prostate adenocarcinomas previously documented by the TCGA Network [25]. Samples were divided by CTCF copy number alteration status to compare RNA expression and DNA methylation. Deletion samples involved over 100,000 kb of the CTCF region. In this set of 333 primary PCs previously annotated by the TCGA Research Network, 27% (90/333) of samples exhibit genomic copy number (CN) loss of *CTCF* (Fig. 4a) with no mutations of *CTCF* detected in the cohort. Samples with CN loss express significantly lower levels of *CTCF* mRNA than tumors diploid for genomic *CTCF* (*P* < 0.03). We analyzed breast cancers in the TCGA in a similar fashion. In 816 breast cancers, we noted 513 (63%) contained deletion, and this correlated with reduced expression (*P* < 0.02; Fig. 4d). These data confirm that CTCF loss is a common finding in primary prostate and breast tumors.

We then analyzed publicly available Illumina Infinium HumanMethylation450 (HM450) data of prostate TCGA specimens for common DNA methylation alterations in CTCF copy number (CN) loss and diploid samples. To minimize confounding variables, we followed quality control procedures as outlined in the initial characterization of these samples by the TCGA Research Network [25]. Samples were grouped based on CTCF CN and diploid tumors contrasted with deep and shallow (combined) deletion samples.

In prostate tumors, a comparison of absolute mean methylation (β-values) in *CTCF* CN loss versus *CTCF* diploid tumors demonstrated 1786 differentially methylated probe sets using a restrictive FDR cut-point (absolute value log2-β-values > 0.5; FDR *q* < 0.01). A volcano plot (Fig. 4b) demonstrates a predominance of hypermethylation events (1684 significant differentially methylated regions (DMR); 94.2%) in tumors harboring loss of *CTCF* CN. In breast cancers, a similar methylation analysis was performed using a FDR *q* < 0.01 (Fig. 4e). There were 670 detected DMRs in CTCF CN loss tumors compared to diploid tumors, and 476 (476/670; 71%) and the majority were hypermethylation events (Fig. 4e). Therefore, CTCF CN loss tumors have increased hypermethylation CTCF intact cancers.

### CTCF deficient prostate and breast cancers tumors contain increased DNA methylation at CTCF sites

CTCF binding sites seem to be a target of altered epigenetic enzymes, as seen in the case of *IDH* mutant gliomas, which exhibit hypermethylation at CTCF-cohesin binding sites [11]. Therefore, we sought to determine whether decreased CTCF expression via CN loss in prostate and breast tumors was principally associated with hypermethylation at CTCF binding sites. Publicly available CTCF ChIP-Seq data in LNCaP was used to define putative prostate CTCF binding sites (GEO: GSE33213) and MCF7 CTCF ChIP-Seq (GEO: GSE30263) employed to define breast cancer CTCF sites. Comparing CTCF binding sites identified by ChIP-Seq against HM450 methylation data of TCGA samples demonstrates a significant enrichment for differentially methylated regions at putative CTCF binding sites (Fig. 4c). Nearly half of all significant hypermethylation (*CTCF* CN loss/*CTCF* diploid) events were located within 2 kb of these CTCF binding sites (889/1786, 49.8%) (Additional file 1: Fig. S6A). In breast cancers, a similar preference for CTCF CN loss tumors to preferentially direct hypermethylation events to CTCF binding sites was seen (Fig. 4f; Additional file 1: Fig. S6B).

In normal human tissues, CTCF binding sites exhibit both highly conserved, but also tissue-specific, DNA binding patterns [10]. Given our findings that CTCF binding sites direct hypermethylation events in solid tumors, we examined whether in breast and prostate tumors this is associated with distinct hypermethylation profiles. A comparison of CTCF ChIP-Seq data from the prostate cell line LNCaP (68,872 binding sites) and the breast line MCF7 (57,936 binding sites) demonstrates that roughly half (35,854) of CTCF binding sites overlap between cells of prostate and breast origin. Using the previously identified differentially methylated regions in CTCF CN deficient tumors from prostate (1786 probes) and breast (670 probes) (Fig. 4b, e), we find 240 prostate and 126 breast CTCF binding sites that are differentially methylated. Only 16 (4%) of CTCF sites showed common hypermethylation in both prostate and breast (Fig. 4g and Additional file 2: Table S2). These data indicate that tumors arising from different tissues display unique CTCF binding patterns that in turn associate with unique tissue-specific methylation patterns.

## Discussion

CTCF is a critical regulator of chromatin organization and cell-type specific gene expression. Despite this characterization, the extent and contribution of CTCF expression in cancer development is not well understood. Our work establishes that loss of CTCF expression plays a pivotal role in spatially directing DNA hypermethylation in human tumors to critical regions of the genome containing CTCF binding sites. Furthermore, the cell-type specific chromatin landscape dictated by CTCF contributes to the variation in tumor suppressor and oncogene function across different tissues and cancer types. Previous reports have documented that normal CTCF binding is crucial for loci-specific maintenance of epigenetic marks at specific critical genes [15, 16, 26, 27]. Collectively, these data suggest epigenetic deregulation by altered CTCF is a crucial mediator of cancer formation and progression.

In the current work, a query of human cancer samples highlights the frequency (27% PCa; 62% BCa) of CTCF copy number alteration in primary tumors (Fig. 4). Adaptation of other publicly available data demonstrates that CTCF copy number status has functional consequences on the DNA methylation landscape. CTCF deletion is specifically associated with increased methylation at CTCF binding sites in breast and prostate tumors. In vitro, we demonstrate that genome-wide DNA methylation alterations occur with short-term reductions (5 days and 10 days) in CTCF protein levels. These data indicate that CTCF downregulation was associated with an increase in hypermethylation events and that these hypermethylation events specifically were enriched for CTCF binding sites in both in vivo and in vitro analyses (Fig. 4c, f).

To date, an instructive mechanism for the targeting of DNA hypermethylation events in aging and cancer has yet to be elucidated. It has been proposed that the evacuation of transcription factors leaves DNA vulnerable to de novo DNA methyltransferase activity (DNMT) [3]. In addition to the physical protection of DNA through its insulator function, CTCF may play an active role in inhibiting DNMT activity through PARP-1 activation [28], suggesting CTCF loss of expression might activate DNMTs also contributing to methylation gain. However, CTCF expression does not correlate with DNMT1, DNMT3A, and DNMT3B in the breast and prostate TCGA datasets (Additional file 1: Fig S7A-F). In the current work, we show extended loss of CTCF binding results in transcriptional silencing and a gain of DNA methylation at promoter-associated CTCF binding sites (Fig. 4). Given the ubiquitous nature of CTCF binding throughout the genome, these data strongly suggest CTCF is important in dictating the cancer-specific DNA methylation landscape. The variation in CTCF sites between tissues of unique origin is well-known [29], and our data suggest CTCF may also explain differences in hypermethylation patterns seen across diverse cancers. A comparison of the overlap of methylated CTCF sites between breast and PCs in the TGCA reveals only 48 CG sites (2%) were common to both cancers (Additional file 2: Table S3) of which 16 also contained CTCF sites (Fig. 4g). Some of the genes associated with these 16 sites included PLEKHA7, AP1M2, ATP9A, and ARVCF known to be downregulated in breast and prostate cancer (data not shown), as well as other cancers [30–35].

Inhibiting DNA methylation induced by CTCF downregulation in vitro using a dose of 5dAza that does not alter proliferation reverses the gene expression changes seen in CTCF deficiency (Fig. 3). This indicates the dependency of a subset of genes on CTCF status. Previous data have demonstrated a role for functional CTCF as a tumor suppressor [36, 37]. However, the mechanisms by which CTCF achieves these effects are diverse and not well understood. Although not a focus of the current work, we did perform pathway analyses of CTCF knockdown and find loss of CTCF negatively regulates a number of pathways, including metabolism, cell motion, hypoxia stress, and hormone response pathways. The androgen receptor contains several CTCF binding sites (within exon 1), and AR expression correlates positively with CTCF expression in the TCGA dataset (Pearson 0.44; *P* = 1.72e−15). Recent work finds that CTCF binding sites are located at the prostate cancer-specific topologically associating domains boundaries for AR loci both in normal and cancer cells which we speculate in human tumor samples may alter expression [38]. No correlation was seen with estrogen receptor expression, and no CTCF sites were noted within the 5’ end of the gene.

## Conclusions

Decreases in CTCF expression alter the DNA methylation landscape in prostate and breast cancer, two of the most common human tumors. These findings are of importance in explaining epigenetic alterations in other tissues and tumor types since CTCF function may be modulated through other mechanisms besides deletion. CTCF binding proteins, including CHD8 [39] and cohesin [40–43], may alter CTCF attachment patterns when their expression is disturbed, a feature that has been noted in a subset of primary PC [44]. In addition, Katinen et al. recently demonstrated CTCF/cohesin binding sites are frequently mutated in cancer, providing another mechanism for disrupting CTCF genome regulation by inhibiting CTCF recognition at specific DNA binding sites [45]. Although the mutational frequency of *CTCF* is low in prostate and breast (1–2%), mutation of *CTCF* occurs at higher rates in other cancers (e.g., 20% in uterine tumors of the TCGA), contributing to alterations in the epigenetic landscape of these cancers. Finally, these data pave the way for the investigation of other proteins with insulator/blocking function in directing accumulated epigenetic alterations with aging and cancer initiation.

## Methods

### Cell lines, plasmids, and antibodies

HPECE6/E7 is one of a series of HPV E6/E7 immortalized cell lines derived from human prostate epithelial cells and cultured as described [22]. The 293FT cells were used in lentiviral packaging. Lentiviral pTRIPz empty vector and non-silencing control (NSC) were purchased from Open Biosystems. Multiple CTCF shRNA sequences were cloned into pTRIPz and tested for efficacy in 293FT cells. Two separate vectors targeting CTCF displaying consistent knockdown by western blot were carried forward (shCTCF1 and shCTCF2). Lentiviral particles were produced by co-transfecting 293FT cells with TransLentiviral shRNA Packaging system and either shCTCF1 (Sequence), shCTCF2 (Sequence), or non-silencing control (NSC) shRNA according to manufacturer’s protocol. Transduction was performed according to manufacturer’s protocols in E6/E7 cells. Stable cell lines were generated via puromycin selection, and shRNA was induced using 2 ug/mL doxycycline. CTCF shRNA and controls were analyzed by western blotting using anti-CTCF rabbit monoclonal antibody (Cell Signaling #3418) to verify knockdown.

Cell counting assays were performed using Hoest staining as previously described [46] to measure response to CTCF knockdown. For 5-aza-2′-deoxycytidine treated plates, 0.2 uM of 5-aza-2′-deoxyazacytidine was administered concurrently with doxycycline beginning at day 5 and maintained throughout the experiment. Media was refreshed every 48 h. Plates were collected and analyzed as described [46]. Average intensity of replicate wells was generated from each plate.

### Methylated DNA immunoprecipitation and array profiling (MeDIP-chip)

The MeDIP-chip approach was adapted from previous studies utilizing methylated DNA immunoprecipitation followed by application to an Affymetrix copy number array (Affymetrix Cytoscan HD) [20, 21]. Biological triplicates of HPECE6/E7 grown for 5 days containing either shNSC, uninduced shCTCF1 (-Dox), or induced shCTCF1 (+Dox) were used for methylation profiling. Genomic DNA depleted of RNA was prepared using DNeasy tissue and blood DNA extraction kit (Qiagen, Hilden, Germany) according to manufacturer’s protocols.

For each DNA sample, 1 ug of genomic DNA was digested with Nsp1 at 37 °C for 2 h followed by adapter ligation at 16 °C for 16 h. Adapter-ligated DNAs were purified using an Amicon Ultra-centrifugation filter (Millipore, Darmstadt, Germany). Purified DNA was then denatured to single strands by heating at 95 °C for 10 min, followed by rapid cooling on ice.

Methylated DNA was immunoprecipitated with 5 ug of anti-5-methylcytosine antibody (Cat. #A3001-200, Zymo Research, Irvine, CA) in IP buffer overnight at 4 °C with rotation. Antibody-DNA complexes were captured with Pierce ChIP-grade Protein A/G magnetic beads (Cat. #26162, ThermoFisher Scientific, Waltham, MA) by rotating at 4 °C for 2 h. Three washes were performed with 0.5 mL of immunoprecipitation buffer. DNA was eluted from the beads with 50 uL of elution buffer (TE + 1% SDS) for 10 min at 65 °C; elution was performed a total of two times, combining eluates. Eluates were Proteinase K treated at 50 °C for 2 h and purified by PCR purification kit (Qiagen).

Whole genome amplification of eluates, fragmentation, array hybridization, and array scanning were performed according to manufacturer’s instructions. CEL files generated from the scanned array image files by the Affymetrix GeneChip Command Console Software were processed using Affymetrix Power Tools. Background subtraction and RMA normalization were performed to obtain normalized log2 transformed raw intensity values. Input subtraction was performed for each IP-input pair for normalization of copy number differences. Statistical analysis was performed on input subtracted values using Limma (R; Bioconductor).

### Methylation analyses including Combined Bisulfite Restriction Analysis (COBRA), MeDIP qPCR, and bisulfite pyrosequencing

Methylation validation was performed using both COBRA and MeDIP-qPCR assays of several regions of the Cytoscan probes (Additional file 2: Table S4). RNA-depleted genomic DNA was isolated using DNeasy DNA isolation kit (Qiagen). One microgram of gDNA was used for bisulfite conversion as previously described [47]. Initially, Combined Bisulfite Restriction Analysis (COBRA) was performed as previously described [48] to validate MeDIP-chip results. Briefly, significant DMRs were analyzed for sequences appropriate for COBRA analysis (TaqI, TCGA or BstUI, CGCG sequence). Primers (Additional file 2: Table S5-6) were designed targeting bisulfite converted DNA and products amplified using PCR. Restriction digest was performed, and resultant products were analyzed by agarose gel electrophoresis. Results were quantified using ImageJ [49].

A second technique, meDIP-qPCR [50], was additionally employed to quantitatively validate the array results and was used in subsequent experiments. MeDIP was performed as described in meDIP-chip methods above with only slight deviations [20, 21]. Primers are listed in Additional file 2: Table S6. For meDIP-qPCR experiments, gDNA was sonicated to obtain fragments < 1000 base pairs, and 4 ug of gDNA was used for each IP using 5 ug of anti-5mC antibody. Purified DNA was then analyzed by qPCR; data are shown as additional file 2: Table S7. Methylation analyses using meDIP-qPCR of CTCF-related genes LTBP2, TNFAIP3, FGF5, EPHA3, and AMIGO2 were performed using primer sequences as listed (Additional file 2: Table S8).

To examine methylation across whole regions quantitatively, bisulfite pyrosequencing was performed as we have described [51]. Bisulfite-modified DNA was then amplified using PCR in preparation for pyrosequencing, with either the forward or reverse primer biotinylated. The biotinylated PCR products were captured with streptavidin sepharose beads, denatured to single strand, and annealed to the sequencing primer for the pyrosequencing assay. SssI methylase-treated bisulfite-converted DNA from human prostate epithelial cell and PCs were used as positive controls, and water substituted for DNA was used as a negative control. Methylation was quantified with the PyroMark MD Pyrosequencing System (Qiagen) within the linear range of the assay. All samples were analyzed by three independent experiments in duplicate.

### Transcriptional profiling and validation

Total RNA was isolated from HPECE6/E7 using PerfectPure RNA Isolation kit (5prime, Hilden, Germany). Sample preparation, quality control, and Human Transcriptome 2.0 Microarray (containing 44,699 genes) profiling were performed according to manufacturer’s protocol (Affymetrix). Affymetrix GeneChip Command Console was used to extract raw data. Transcriptome Analysis Console (v3.0 Affymetrix) was used for RMA normalization and statistical analysis. Genes with FDR < 10% were considered significant. Biological validation of significant genes was performed in biological replicates in both independent vectors targeting CTCF (shCTCF1 and shCTCF2) as well as non-silencing shRNA oby qPCR. Heat maps were created using *Z*-score transformation of normalized intensity values using ggplot2 (Bioconductor) in R. Gene ontology was performed using DAVID (Nature protocols 2009 citation—see website). Significant up- and downregulated genes are available online.

### Chromatin immunoprecipitation (ChIP)

Decreases in CTCF binding after shCTCF induction were verified by chromatin immunoprecipitation. Potential CTCF binding sites were identified using ENCODE CTCF ChIP-Seq data from UCSC for highly conserved CTCF binding sites and LNCaP CTCF ChIP-Seq data for potential prostate specific CTCF binding sites. The ChIP assay was performed as previously described [52]. Briefly, 1 × 10^7 cells were used for each IP with 5 ug of anti-CTCF antibody (Cell Signaling #3418) at 4 °C overnight. Negative controls performed using anti-Rabbit IgG antibody (Cell Signaling #3900). Immunoprecipitation complexes were purified with Protein A/G magnetic beads (Cat. #26162, ThermoFisher Scientific) at 4 °C for 2 h, followed by washing. Elution was performed twice, using 75 uL elution buffer (1% SDS, 100 mM NaHCO3), combining eluates for a total of 150 uL. Crosslink reversal was performed overnight at 67 °C, followed by RNase and Proteinase K treatment. DNA was purified using PCR purification kit (Qiagen). Purified DNA was analyzed by qPCR.

### Quantitative polymerase chain reaction (qPCR)

For gene expression analysis, DNase treated total RNA from cultured cells was isolated using PerfectPure RNA isolation kit (5prime). A total of 2 ug of RNA was used for reverse transcription using qScript cDNA Synthesis Kit (Quantabio, Beverly, MA). For ChIP and meDIP analysis, column purified DNA was used for analysis. Quantitative PCR was performed using PerfeCTa SYBR Green FastMix (Quantabio, Beverly, MA) on a Bio-Rad CFX96 (Bio-Rad, Hercules, CA) using experimental samples including no reverse transcriptase and cDNA free negative controls.

### TCGA analysis and CTCF Chip-Seq in LNCaP

Level 3 RNA-sequencing, GISTIC2 copy number alterations, and HM450 methylation data were downloaded for 333 primary prostate adenocarcinomas previously documented by the TCGA Network [25]. Samples were divided by CTCF copy number alteration status to compare RNA expression and DNA methylation. Deletion samples involved over 100,000 kb of the CTCF region. Analyses were conducted in R using TCGA biolinks package from Bioconductor. Boxplots and volcano plots are created using ggplot2 (Bioconductor) in R.

### Bioinformatics analysis

#### MeDIP-chip

We followed guidelines for methylation analysis as outlined previously for MeDIP-chip analysis using Affymetrix genotyping and copy number arrays [20, 21]. Using Affymetrix Cytoscan HD probe annotation data, we matched array probes to their predicted *NspI* digested fragments to predict enrichment regions. Analysis was restricted only to those fragments containing CpG sequences as defined by CpG density > 2.5% [20, 21]. We considered *P* values < 0.01 as significant; several probe regions identified by this cut point were further validated using Combined Bisulfite Restriction Analysis (COBRA) assay and MeDIP-qPCR (Additional file 1: Fig. S1).

#### Motif finding

Searching methylated sequences for known transcription factor binding sites (TFBS) was performed using the HOMER package [23]. Significant hypermethylated or hypomethylated sequences were searched for known TFBS using the de novo motif finding parameters. Cytoscan HD array targeted *NspI* fragments containing CpG sequences were used as background sequences.

#### Annotation data

Hg19 genome annotation data was obtained from Ensembl. CTCF binding sites for prostate cells were obtained from CTCF ChIP-Seq in LNCaP cells (GEO: GSE33213). For breast cancer data, CTCF binding sites were obtained from CTCF ChIP-Seq in MCF7 cells (GSE30263). Transcribed regions including introns, exons and untranslated regions, CpG island locations, and CTCF binding sites were matched to Cytoscan HD array *NspI* fragment or Illumina 450k Methylation array probe genomic location using BedTools.

## Supplementary information

**Additional file 1: Figure S1.** Combined Bisulfite Restriction Analysis (COBRA) and MeDIP-qPCR Validation of Six Differentially Methylated Identified Probes Two different methods were used to validate probes identified by MeDIP-chip analysis in biological replicates after 5 day shRNA induction. Detailed information of array probes (Table S2), COBRA characteristics (Table S3), and MeDIP-chip vs. MeDIP-qPCR comparisons (Table S4) are provided. We show that methylation levels in the original DNA sample are represented by the relative amounts of digested and undigested PCR product in a linearly quantitative fashion. MeDIP-qPCR results are presented as mean+SD of technical triplicates (**P<0.01, *P<0.05). Arrows denote uncut bands. (*A*) COBRA using *TaqI* (left) and MeDIP-qPCR (right) of Cytoscan HD probe C-4QPFF region. (*B*) COBRA using *TaqI* (left) and MeDIP-qPCR (right) of Cytoscan HD probe C-6LWFW region. (*C*) COBRA using *TaqI* (left) and MeDIP-qPCR (right) of Cytoscan HD probe C-4QXQN region. (*D*) COBRA using *TaqI* (left) and MeDIP-qPCR (right) of Cytoscan HD probe C-3GEQV region. (*E*) COBRA using *BstUI* restriction enzyme of Cytoscan HD probe C-3GEQV region. (F) COBRA using *TaqI* (left) and MeDIP-qPCR (right) of Cytoscan HD probe C-6XOOB region. **Figure S2.** Quantitative PCR Validation of Differentially Expressed Genes Identified by Transcriptional Array Following CTCF Knockdown (*A*) Six genes identified by transcriptional array profiling as down regulated following CTCF knockdown were validated using qPCR in biological replicates. Significant downregulation was confirmed in two independent shRNAs targeting CTCF following 5 days of doxycycline induction. Data shown are mean±SD of technical triplicates representative of multiple experiments (**P < 0.01, *P < 0.05). (*B*) Three genes identified by transcriptional array profiling as up regulated following CTCF knockdown were validated using qPCR in biological replicates. Significant upregulation was confirmed in two independent shRNAs targeting CTCF following 5 days of doxycycline induction. Data show are mean±SD of technical triplicates representative of multiple experiments (**P < 0.01, *P < 0.05). **Figure S3.** Expanded Results of ChIP-qPCR and meDIP-qPCR in Immortalized HPECs Following 10 days of shRNA Induction (*A*) ChIP-qPCR Results for *TNFAIP3* promoter CTCF binding site. (*B*) ChIP-qPCR Results for *FGF5* promoter CTCF binding site. (*C*) ChIP-qPCR Results for *AMIGO2* promoter CTCF binding sites. (*D*) ChIP-qPCR Results for *EPHA3* promoter CTCF binding sites. (*E*) MeDIP-qPCR Results for *TNFAIP3* promoter CTCF binding sites. (*F*) MeDIP-qPCR Results for *FGF5* promoter CTCF binding site. (*G*) MeDIP-qPCR Results for *AMIGO2* promoter CTCF binding sites. (*H*) MeDIP-qPCR Results for *EPHA3* promoter CTCF binding sites. All data are presented mean ±SD of technical triplicates, one representative experiment of three. *P < 0.05; **P < 0.01. **Figure S4.** Methylation Quantification of *LTBP2* Promoter CTCF binding site by Quantitative Pyrosequencing of Bisulfite Converted DNA to Validate meDIP-qPCR Results (*A*) Methylation quantification of HPECs +Dox in shCTCF2 after 10 days of shRNA induction (Data represent mean+SE of two independent experiments). (*B*) Methylation quantification of HPECs +Dox in shNSC after 10 days of shRNA induction (Data represent mean+SE of two independent experiments). (*C*) MeDIP-qPCR demonstrating increased methylation at *LTBP2* promoter CTCF binding site after 10 days of shCTCF induction. Data shown are mean+SD of technical triplicates from one representative experiment of three. *P < 0.05 and *P < 0.01. **Figure S5.** Correlation analysis of promoter methylation vs. mRNA expression in TCGA prostate cancer samples. Methylation B-values and mRNA (log2 RSEM) expression levels compared for (*A*) LTBP2, (*B*) TNFAIP3, (*C*) FGF5, (*D*) EPHA3, and (*E*) AMIGO2 genes. Pearson correlation *R*-value shown. Data was downloaded from cBioPortal for PRAD TCGA samples (Cell 2015). **Figure S6.** Comparison of hypermethylated versus hypomethylated probes with proximity to CTCF binding sites in TCGA tumor samples (*A*) Prostate cancer cell line LNCaP CTCF ChIP-Seq (GSE33213) identified putative CTCF binding sites. The percentage of hypermethylated probes and percentage of hypomethylated probes were calculated with respect to proximity to CTCF binding sites. (*B*) Breast cancer cell line MCF7 CTCF ChIP-Seq (GSE30263) identified putative CTCF binding sites. The percentage of hypermethylated probes and percentage of hypomethylated probes were calculated with respect to proximity to CTCF binding sites. **Figure S7.** Correlation analysis of CTCF mRNA expression vs. DNMTs or AR/ER mRNA expression in TCGA prostate or breast cancer samples. CTCF mRNA (log2 RSEM) expression levels compared for (*A*) DNMT1, (*B*) DNMT3A, (*C*) DNMT3B in prostate cancer; (*D*) DNMT1, (*E*) DNMT3A, (*F*) DNMT3B in breast cancer; (G) AR in prostate cancer and (H) ER in breast cancer. Pearson correlation *R*-value and p-values are shown. Data was downloaded from cBioPortal for PRAD TCGA samples (Cell 2015).

**Additional file 2: Table S1.** Significantly altered genes that contain a CTCF binding site in the TSS and significant hyper- or hypomethylation on methylation array. **Table S2:** Common hypermethylation CGs containing CTCF binding sites in both prostate and breast tumors. **Table S3.** Differentially methylated CGs in both prostate and breast tumors. **Table S4:** Cytoscan Probes’ Characteristics Used in Array Validation. **Table S5:** Combined Bisulfite Restriction Analysis (COBRA) Assay Characteristics (Related to Additional file 1: Fig. S1). **Table S6:** Primer sequences of COBRA and MeDIP-qPCR used for Validation of Cytoscan Array (Related to Additional file 1: Fig. S1). **Table S7:** MeDIP-qPCR Performed region and methylation values after CTCF knockdown. **Table S8:** Primers Used for ChIP-qPCR and MeDIP-qPCR in Extended Knockdown Studies (Related to Fig.3 and Additional file 1: Fig. S3)

## Data Availability

All data generated or analyzed during this study are included in this published article and its supplementary information files. Two Microarrays’ raw data and processed data used in this study have been deposited on GEO, accession numbers GSE93328 and GSE93363.

## Abbreviations

CTCF: CCCTC-binding factor
TNFAIP3: TNF alpha induced protein 3
FGF5: Fibroblast growth factor 5
EPHA3: EPH receptor A3
AMIGO2: Adhesion molecule with Ig like domain 2
LTBP2: Latent transforming growth factor beta binding protein 2
COBRA: Combined Bisulfite Restriction Analysis
MeDIP: Methylated DNA immunoprecipitation
DMRs: Differentially methylated regions
