# Supplementary material for: CTCF loss mediates unique DNA hypermethylation landscapes in human cancers
Source: Clin Epigenetics. 2020 Jun 5;12:80. doi: 10.1186/s13148-020-00869-7 (PMC7275597; doi:10.1186/s13148-020-00869-7)
Supplement: Supplementary file 1 — Additional file 1: Figure S1. Combined Bisulfite Restriction Analysis (COBRA) and MeDIP-qPCR Validation of Six Differentially Methylated Identified Probes Two different methods were used to validate probes identified by MeDIP-chip analysis in biological replicates after 5 day shRNA induction. Detailed information of array probes (Table S2), COBRA characteristics (Table S3), and MeDIP-chip vs. MeDIP-qPCR comparisons (Table S4) are provided. We show that methylation levels in the original DNA sample are represented by the relative amounts of digested and undigested PCR product in a linearly quantitative fashion. MeDIP-qPCR results are presented as mean+SD of technical triplicates (**P<0.01, *P<0.05). Arrows denote uncut bands. (A) COBRA using TaqI (left) and MeDIP-qPCR (right) of Cytoscan HD probe C-4QPFF region. (B) COBRA using TaqI (left) and MeDIP-qPCR (right) of Cytoscan HD probe C-6LWFW region. (C) COBRA using TaqI (left) and MeDIP-qPCR (right) of Cytoscan HD probe C-4QXQN region. (D) COBRA using TaqI (left) and MeDIP-qPCR (right) of Cytoscan HD probe C-3GEQV region. (E) COBRA using BstUI restriction enzyme of Cytoscan HD probe C-3GEQV region. (F) COBRA using TaqI (left) and MeDIP-qPCR (right) of Cytoscan HD probe C-6XOOB region. Figure S2. Quantitative PCR Validation of Differentially Expressed Genes Identified by Transcriptional Array Following CTCF Knockdown (A) Six genes identified by transcriptional array profiling as down regulated following CTCF knockdown were validated using qPCR in biological replicates. Significant downregulation was confirmed in two independent shRNAs targeting CTCF following 5 days of doxycycline induction. Data shown are mean±SD of technical triplicates representative of multiple experiments (**P < 0.01, *P < 0.05). (B) Three genes identified by transcriptional array profiling as up regulated following CTCF knockdown were validated using qPCR in biological replicates. Significant upregulation was confirmed in two independent shRNAs t [file 13148_2020_869_MOESM1_ESM.docx]

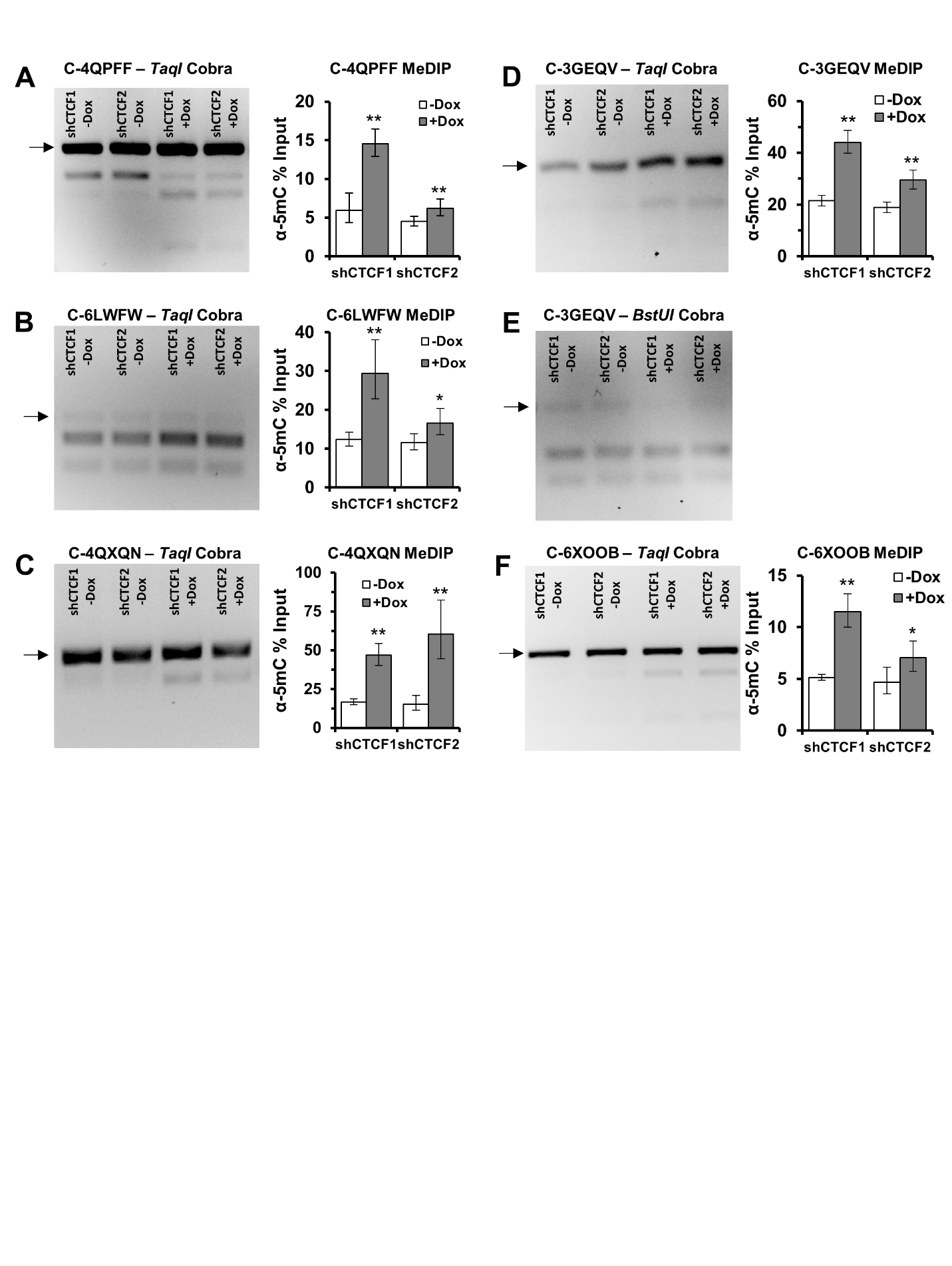


**Fig. S1. Combined Bisulfite Restriction Analysis (COBRA) and MeDIP-qPCR Validation of Six Differentially Methylated Identified Probes** Two different methods were used to validate probes identified by MeDIP-chip analysis in biological replicates after 5 day shRNA induction. Detailed information of array probes (Table S2), COBRA characteristics (Table S3), and MeDIP-chip vs. MeDIP-qPCR comparisons (Table S4) are provided. We show that methylation levels in the original DNA sample are represented by the relative amounts of digested and undigested PCR product in a linearly quantitative fashion. MeDIP-qPCR results are presented as mean+SD of technical triplicates (***P*<0.01, **P*<0.05). Arrows denote uncut bands. (*A*) COBRA using *TaqI* (left) and MeDIP-qPCR (right) of Cytoscan HD probe C-4QPFF region. (*B*) COBRA using *TaqI* (left) and MeDIP-qPCR (right) of Cytoscan HD probe C-6LWFW region. (*C*) COBRA using *TaqI* (left) and MeDIP-qPCR (right) of Cytoscan HD probe C-4QXQN region. (*D*) COBRA using *TaqI* (left) and MeDIP-qPCR (right) of Cytoscan HD probe C-3GEQV region. (*E*) COBRA using *BstUI* restriction enzyme of Cytoscan HD probe C-3GEQV region. (F) COBRA using *TaqI* (left) and MeDIP-qPCR (right) of Cytoscan HD probe C-6XOOB region.


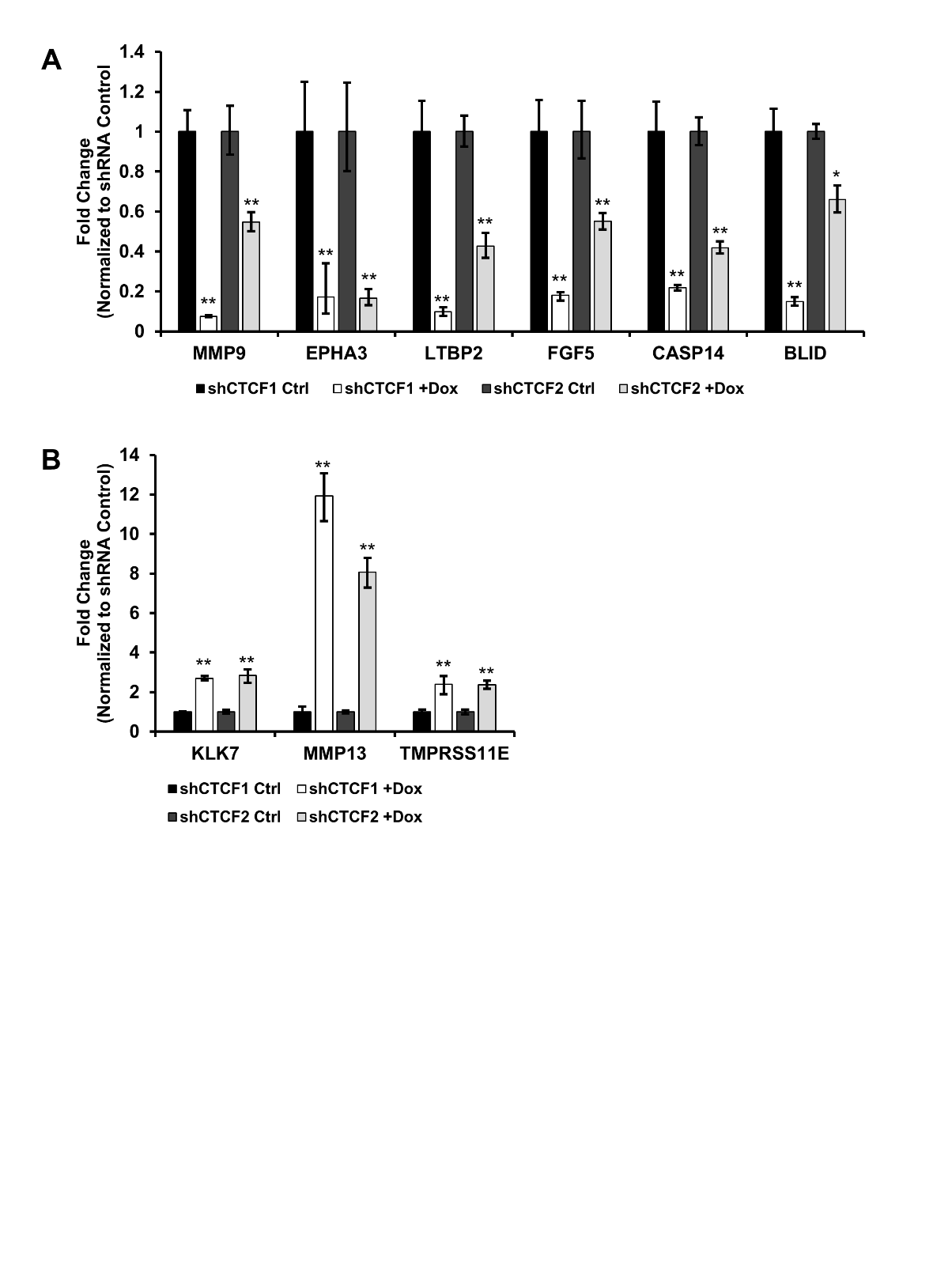


**Fig. S2. Quantitative PCR Validation of Differentially Expressed Genes Identified by Transcriptional Array Following CTCF Knockdown** (*A*) Six genes identified by transcriptional array profiling as down regulated following CTCF knockdown were validated using qPCR in biological replicates. Significant downregulation was confirmed in two independent shRNAs targeting CTCF following 5 days of doxycycline induction. Data shown are mean+SD of technical triplicates representative of multiple experiments (***P* < 0.01, **P* < 0.05). (*B*) Three genes identified by transcriptional array profiling as up regulated following CTCF knockdown were validated using qPCR in biological replicates. Significant upregulation was confirmed in two independent shRNAs targeting CTCF following 5 days of doxycycline induction. Data show are mean+SD of technical triplicates representative of multiple experiments (***P* < 0.01, **P* < 0.05).


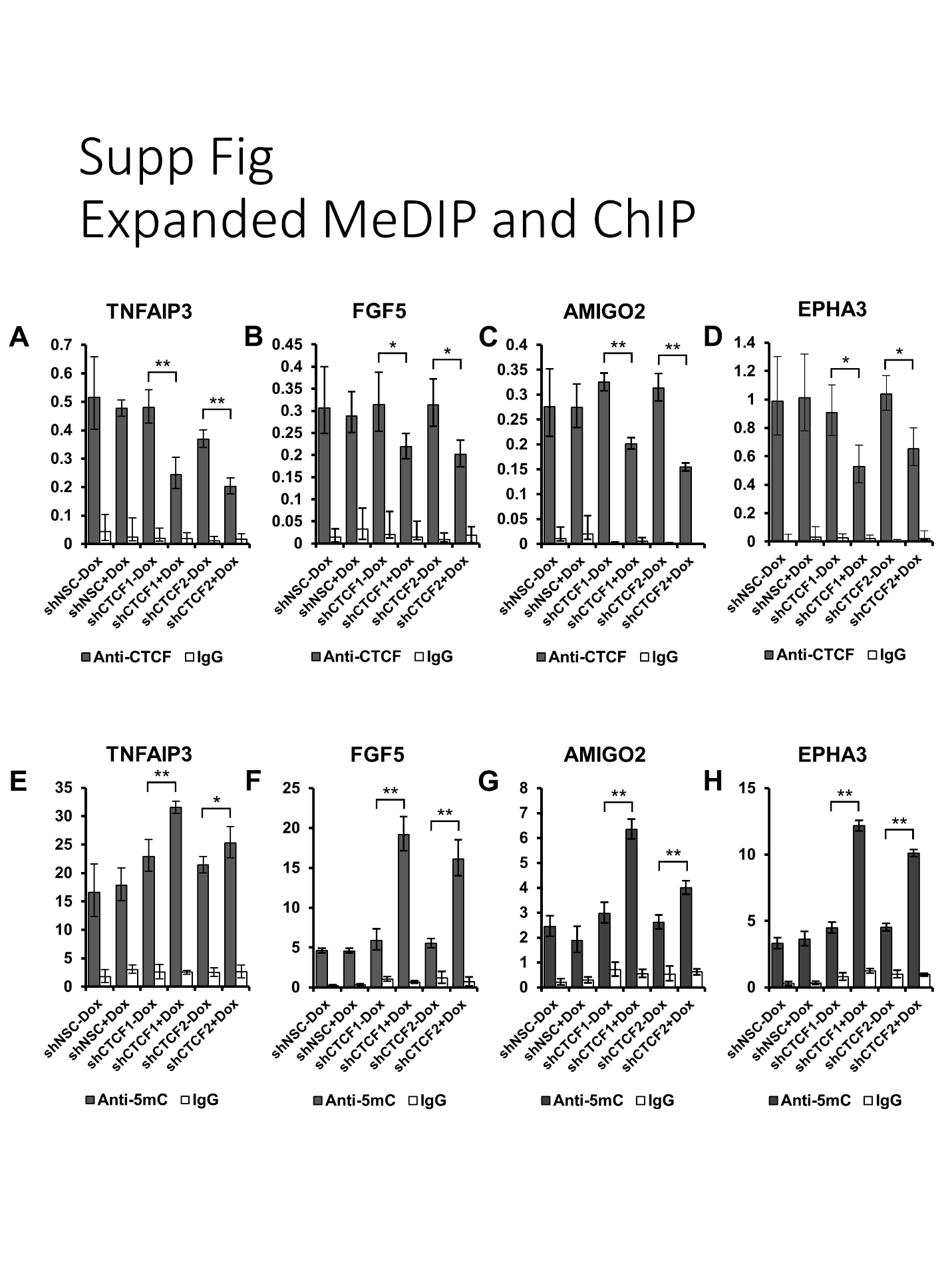


**Fig. S3. Expanded Results of ChIP-qPCR and meDIP-qPCR in Immortalized HPECs Following 10 days of shRNA Induction** (*A*) ChIP-qPCR Results for *TNFAIP3* promoter CTCF binding site. (*B*) ChIP-qPCR Results for *FGF5* promoter CTCF binding site. (*C*) ChIP-qPCR Results for *AMIGO2* promoter CTCF binding sites. (*D*) ChIP-qPCR Results for *EPHA3* promoter CTCF binding sites. (*E*) MeDIP-qPCR Results for *TNFAIP3* promoter CTCF binding sites. (*F*) MeDIP-qPCR Results for *FGF5* promoter CTCF binding site. (*G*) MeDIP-qPCR Results for *AMIGO2* promoter CTCF binding sites. (*H*) MeDIP-qPCR Results for *EPHA3* promoter CTCF binding sites. All data are presented mean +SD of technical triplicates, one representative experiment of three. **P* < 0.05; ***P* < 0.01.

**Fig. S4. Methylation Quantification of *LTBP2* Promoter CTCF binding site by Quantitative Pyrosequencing of Bisulfite Converted DNA to Validate meDIP-qPCR Results** (*A*) Methylation quantification of HPECs +Dox in shCTCF2 after 10 days of shRNA induction (Data represent mean+SE of two independent experiments). (*B*) Methylation quantification of HPECs +Dox in shNSC after 10 days of shRNA induction (Data represent mean+SE of two independent experiments). (*C*) MeDIP-qPCR demonstrating increased methylation at *LTBP2* promoter CTCF binding site after 10 days of shCTCF induction. Data shown are mean+SD of technical triplicates from one representative experiment of three. **P* < 0.05 and **P* < 0.01.


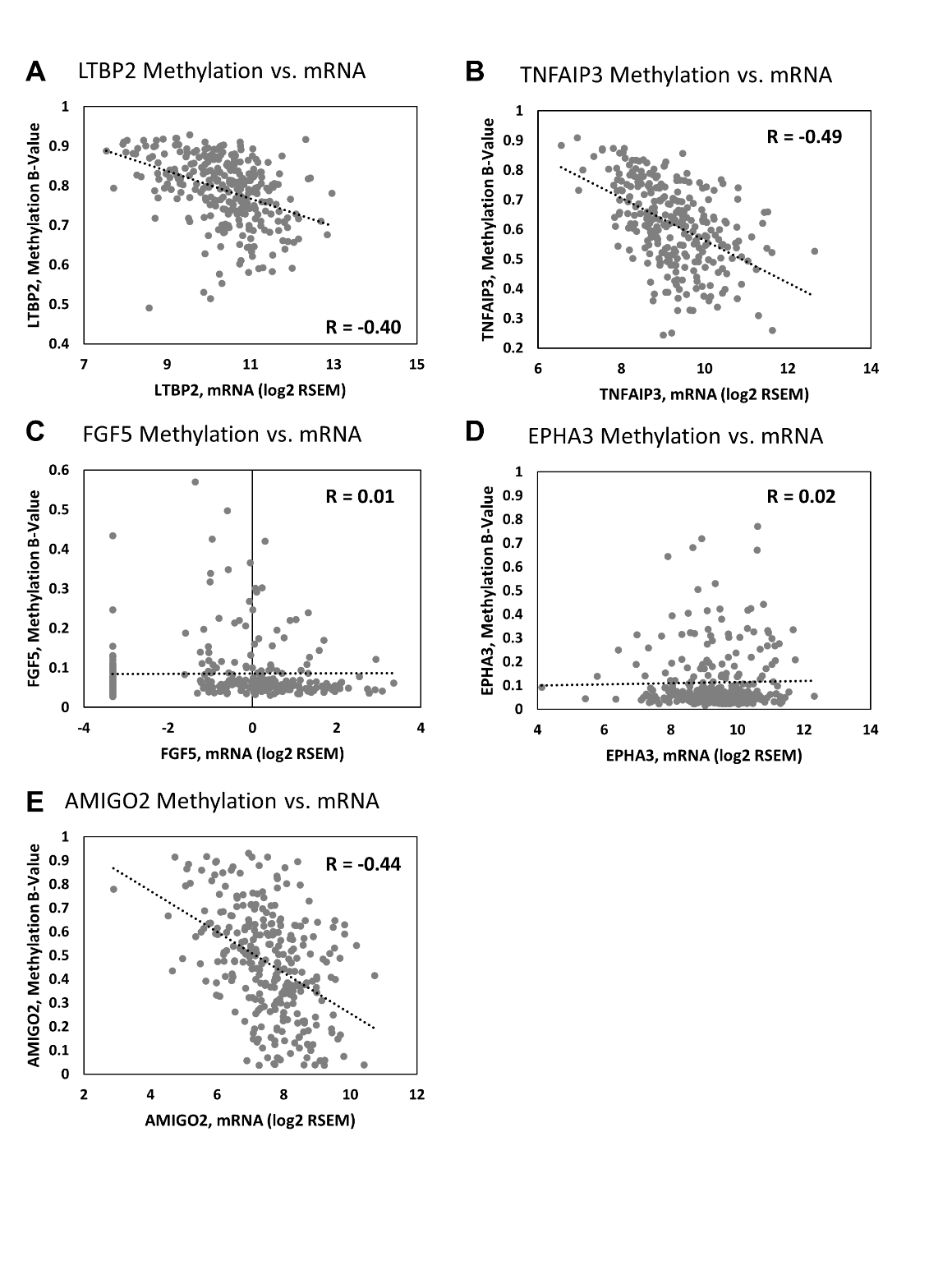


**Fig. S5. Correlation analysis of promoter methylation vs. mRNA expression in TCGA prostate cancer samples.** Methylation B-values and mRNA (log2 RSEM) expression levels compared for (*A*) LTBP2, (*B*) TNFAIP3, (*C*) FGF5, (*D*) EPHA3, and (*E*) AMIGO2 genes. Pearson correlation *R*-value shown. Data was downloaded from cBioPortal for PRAD TCGA samples (Cell 2015).


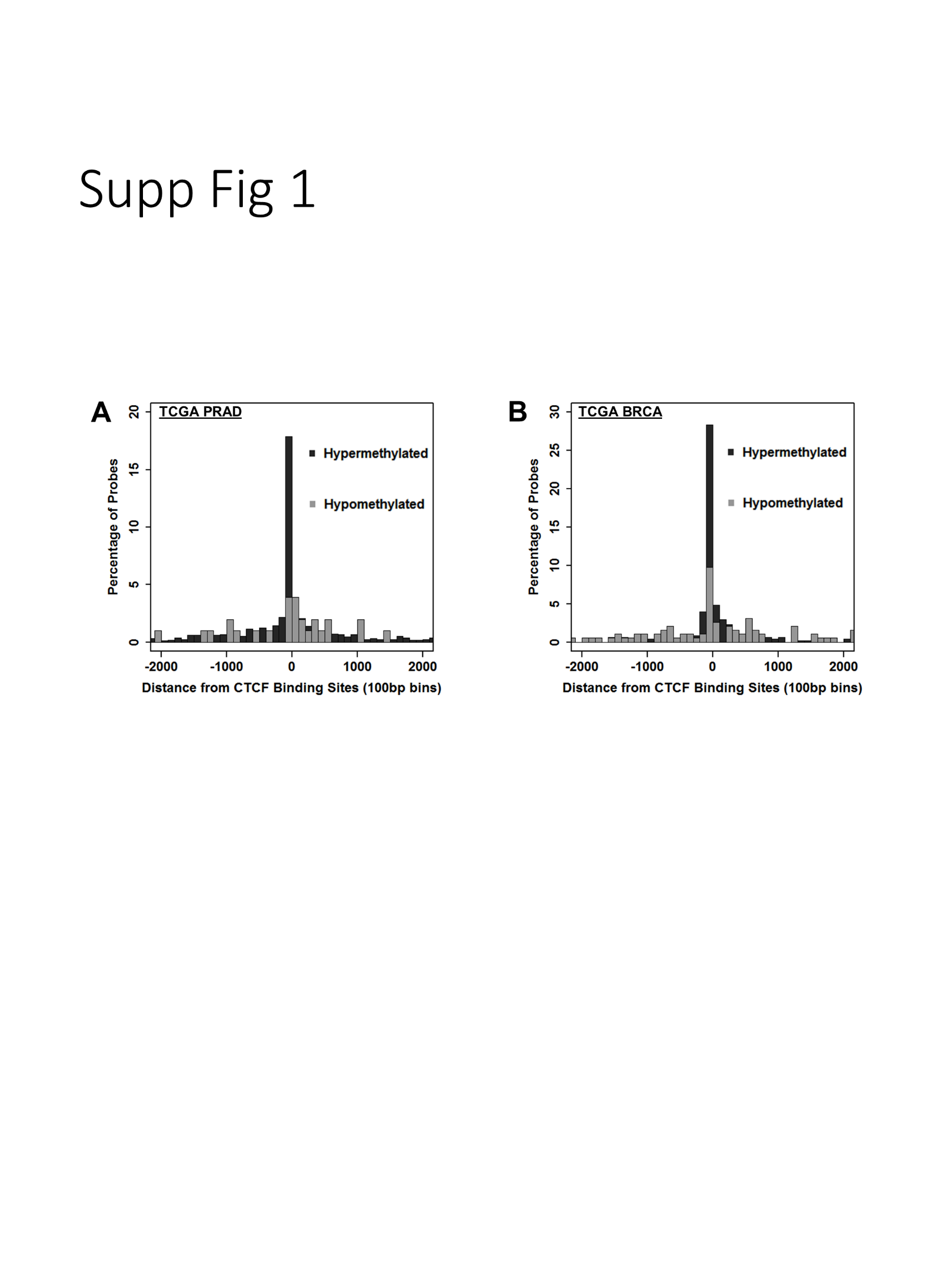


**Fig. S6. Comparison of hypermethylated versus hypomethylated probes with proximity to CTCF binding sites in TCGA tumor samples** (*A*) Prostate cancer cell line LNCaP CTCF ChIP-Seq (GSE33213) identified putative CTCF binding sites. The percentage of hypermethylated probes and percentage of hypomethylated probes were calculated with respect to proximity to CTCF binding sites. (*B*) Breast cancer cell line MCF7 CTCF ChIP-Seq (GSE30263) identified putative CTCF binding sites. The percentage of hypermethylated probes and percentage of hypomethylated probes were calculated with respect to proximity to CTCF binding sites.

**Fig. S7. Correlation analysis of CTCF mRNA expression vs. DNMTs or AR/ER mRNA expression in TCGA prostate or breast cancer samples.** CTCF mRNA (log2 RSEM) expression levels compared for (*A*) DNMT1, (*B*) DNMT3A, (*C*) DNMT3B in prostate cancer; (*D*) DNMT1, (*E*) DNMT3A, (*F*) DNMT3B in breast cancer; (G) AR in prostate cancer and (H) ER in breast cancer. Pearson correlation *R*-value and p-values are shown. Data was downloaded from cBioPortal for PRAD TCGA samples (Cell 2015).
