# Supplementary material for: CTCF loss mediates unique DNA hypermethylation landscapes in human cancers
Source: Clin Epigenetics. 2020 Jun 5;12:80. doi: 10.1186/s13148-020-00869-7 (PMC7275597; doi:10.1186/s13148-020-00869-7)
Supplement: Supplementary file 2 — Additional file 2: Table S1. Significantly altered genes that contain a CTCF binding site in the TSS and significant hyper- or hypomethylation on methylation array. Table S2: Common hypermethylation CGs containing CTCF binding sites in both prostate and breast tumors. Table S3. Differentially methylated CGs in both prostate and breast tumors. Table S4: Cytoscan Probes’ Characteristics Used in Array Validation. Table S5: Combined Bisulfite Restriction Analysis (COBRA) Assay Characteristics (Related to Additional file 1: Fig. S1). Table S6: Primer sequences of COBRA and MeDIP-qPCR used for Validation of Cytoscan Array (Related to Additional file 1: Fig. S1). Table S7: MeDIP-qPCR Performed region and methylation values after CTCF knockdown. Table S8: Primers Used for ChIP-qPCR and MeDIP-qPCR in Extended Knockdown Studies (Related to Fig.3 and Additional file 1: Fig. S3) [file 13148_2020_869_MOESM2_ESM.docx]

**Additional file 2: SUPPLEMENTAL TABLES (DAMASHKE ET AL.)**

**Table S1: Significantly altered genes that contain a CTCF binding site in the TSS and significant hyper- or hypomethylation on methylation array.**

|  | **Increased methylation and Decreased Expression (n=147)** | | |  | |  | | **Decreased methylation and Increased Expression (n=102)** | |
| --- | --- | --- | --- | --- | --- | --- | --- | --- | --- |
| **Gene Symbol** | **Methylation FC*** | | **Expression FC** | |  | | **Gene Symbol** | **Methylation FC** | **Expression FC** |
| AAED1 | 1.35 | 0.89 | |  | | ABHD17C | | 0.79 | 1.30 |
| ABR | 1.76 | 0.83 | |  | | ABHD2 | | 0.60 | 1.21 |
| ACLY | 1.64 | 0.90 | |  | | ACBD7 | | 0.53 | 1.05 |
| ACOX3 | 1.40 | 0.86 | |  | | ACVR1B | | 0.65 | 1.08 |
| ADAMTS12 | 1.46 | 0.76 | |  | | ADD3 | | 0.52 | 1.23 |
| ADM2 | 2.01 | 0.75 | |  | | AKAP7 | | 0.64 | 1.08 |
| ADRB2 | 1.53 | 0.87 | |  | | BIRC5 | | 0.95 | 1.17 |
| ADTRP | 1.55 | 0.91 | |  | | BLNK | | 0.90 | 1.23 |
| AEBP1 | 1.56 | 0.81 | |  | | BNIP3 | | 0.46 | 1.08 |
| AMIGO2 | 2.13 | 0.58 | |  | | BRCA1 | | 0.65 | 1.19 |
| APBB2 | 2.74 | 0.73 | |  | | C11orf58 | | 0.64 | 1.11 |
| ARSJ | 1.91 | 0.88 | |  | | C14orf93 | | 0.73 | 1.07 |
| ATP2B1 | 1.50 | 0.89 | |  | | CBY1 | | 0.80 | 1.15 |
| BEND7 | 1.32 | 0.80 | |  | | CCDC134 | | 0.92 | 1.08 |
| BTBD10 | 1.66 | 0.91 | |  | | CFI | | 0.55 | 1.08 |
| BTBD9 | 1.36 | 0.95 | |  | | CLUAP1 | | 0.66 | 1.13 |
| C10orf105 | 1.37 | 0.97 | |  | | COX8A | | 0.83 | 1.16 |
| C3 | 1.71 | 0.79 | |  | | CRCT1 | | 0.69 | 1.21 |
| C4orf26 | 1.72 | 0.69 | |  | | DAG1 | | 0.70 | 1.10 |
| CACNB3 | 1.71 | 0.90 | |  | | DBNDD1 | | 0.69 | 1.12 |
| CASP14 | 1.64 | 0.59 | |  | | DHRS4L2 | | 0.65 | 1.07 |
| CCNB1IP1 | 1.77 | 0.77 | |  | | DNAJA3 | | 0.63 | 1.07 |
| CD48 | 1.51 | 0.92 | |  | | DOPEY2 | | 0.63 | 1.04 |
| CDC14C | 1.39 | 0.88 | |  | | DPF2 | | 0.50 | 1.20 |
| CDK14 | 1.61 | 0.65 | |  | | ETV7 | | 0.73 | 1.10 |
| CDK6 | 1.50 | 0.92 | |  | | GALNT7 | | 0.67 | 1.31 |
| CDR1 | 1.18 | 0.85 | |  | | GPR183 | | 0.79 | 1.13 |
| CLTB | 2.35 | 0.90 | |  | | GRB7 | | 0.74 | 1.05 |
| CLTCL1 | 2.19 | 0.95 | |  | | GSTM1 | | 0.75 | 1.05 |
| COG3 | 1.10 | 0.89 | |  | | HOMEZ | | 0.66 | 1.08 |
| COL4A3 | 2.48 | 0.90 | |  | | HOXA3 | | 0.73 | 1.08 |
| CORO2B | 2.09 | 0.88 | |  | | IFIT3 | | 0.85 | 1.27 |
| CSNK2A2 | 1.54 | 0.89 | |  | | IGFBP2 | | 0.89 | 1.19 |
| CSRNP2 | 1.41 | 0.90 | |  | | ITPR1 | | 0.68 | 1.11 |
| CUL4A | 1.42 | 0.85 | |  | | KATNB1 | | 0.54 | 1.05 |
| CUL4B | 1.63 | 0.79 | |  | | LINC01123 | | 0.48 | 1.11 |
| CXCL11 | 1.22 | 0.83 | |  | | LMNA | | 0.73 | 1.09 |
| CXorf40B | 1.35 | 0.93 | |  | | LOC100286922 | | 0.69 | 1.07 |
| DBNL | 1.73 | 0.95 | |  | | LOC100288798 | | 0.47 | 1.09 |
| DENND5B | 1.22 | 0.89 | |  | | LOC339803 | | 0.59 | 1.12 |
| DERL2 | 1.46 | 0.89 | |  | | LOC644554 | | 0.67 | 1.04 |
| DES | 1.44 | 0.97 | |  | | LOXL1 | | 0.47 | 1.11 |
| DFNA5 | 1.34 | 0.86 | |  | | LPAR6 | | 0.56 | 1.04 |
| DIXDC1 | 1.33 | 0.94 | |  | | LRRC8B | | 0.63 | 1.24 |
| DMD | 2.14 | 0.89 | |  | | LSM10 | | 0.81 | 1.05 |
| DNAJC12 | 1.57 | 0.87 | |  | | LSM3 | | 0.59 | 1.12 |
| DYM | 1.70 | 0.89 | |  | | LYRM9 | | 0.85 | 1.06 |
| EBLN2 | 1.17 | 0.82 | |  | | MAP2K3 | | 0.76 | 1.02 |
| EIF4G3 | 2.22 | 0.90 | |  | | MAPK13 | | 0.70 | 1.06 |
| EPHA3 | 1.76 | 0.91 | |  | | MFF | | 0.78 | 1.05 |
| ERCC6 | 1.36 | 0.84 | |  | | MMP13 | | 0.96 | 3.85 |
| ETV5 | 2.20 | 0.79 | |  | | MOV10 | | 0.57 | 1.11 |
| FBXW11 | 1.11 | 0.84 | |  | | MRPL10 | | 0.71 | 1.20 |
| FDFT1 | 1.68 | 0.90 | |  | | MTPAP | | 0.52 | 1.03 |
| FGF5 | 2.07 | 0.68 | |  | | MTUS1 | | 0.78 | 1.30 |
| FSTL1 | 1.37 | 0.79 | |  | | MTX2 | | 0.52 | 1.32 |
| GJB5 | 1.92 | 0.84 | |  | | MXI1 | | 0.64 | 1.10 |
| GLB1 | 1.47 | 0.95 | |  | | MYB | | 0.44 | 1.54 |
| GNA13 | 1.59 | 0.96 | |  | | NCAPD2 | | 0.66 | 1.30 |
| GPAM | 1.46 | 0.78 | |  | | NDUFA5 | | 0.82 | 1.15 |
| GRAMD3 | 1.84 | 0.80 | |  | | NHLH2 | | 0.62 | 1.14 |
| GTF2IRD2 | 1.86 | 0.92 | |  | | NSMAF | | 0.91 | 1.10 |
| H2AFY2 | 1.89 | 0.94 | |  | | OCLN | | 0.76 | 1.30 |
| HADHA | 1.37 | 0.93 | |  | | OTUD7B | | 0.83 | 1.05 |
| HAUS2 | 1.53 | 0.84 | |  | | PARD6B | | 0.70 | 1.17 |
| HCFC2 | 1.46 | 0.87 | |  | | PI4KAP1 | | 0.84 | 1.16 |
| IL1RAP | 1.94 | 0.70 | |  | | PIK3C2B | | 0.77 | 1.41 |
| ITM2C | 1.54 | 0.85 | |  | | PLEKHA6 | | 0.45 | 1.22 |
| JAG1 | 1.62 | 0.86 | |  | | POU5F1P4 | | 0.68 | 1.08 |
| KCNJ15 | 1.68 | 0.68 | |  | | PSORS1C2 | | 0.72 | 1.02 |
| KCTD11 | 1.47 | 0.90 | |  | | PTCH1 | | 0.77 | 1.31 |
| KIF3A | 1.42 | 0.90 | |  | | PTPRH | | 0.51 | 1.16 |
| LAMA3 | 1.38 | 0.92 | |  | | RAB9A | | 0.75 | 1.05 |
| LAMP2 | 2.26 | 0.85 | |  | | RHOF | | 0.63 | 1.06 |
| LIMK2 | 1.19 | 0.80 | |  | | RNU6-71P | | 0.93 | 1.13 |
| LINC00844 | 1.12 | 0.60 | |  | | RUNX2 | | 0.89 | 1.43 |
| LINC01234 | 1.54 | 0.91 | |  | | SEPN1 | | 0.72 | 1.19 |
| LINCR-0003 | 2.14 | 0.87 | |  | | SLC12A6 | | 0.74 | 1.24 |
| LIPG | 1.46 | 0.67 | |  | | SLC22A18 | | 0.75 | 1.13 |
| LOC100506834 | 1.66 | 0.93 | |  | | SLC38A3 | | 0.55 | 1.10 |
| LOC101927636 | 1.45 | 0.88 | |  | | SLC43A2 | | 0.52 | 1.11 |
| LOC101928437 | 1.36 | 0.92 | |  | | SLC7A5P1 | | 0.72 | 1.11 |
| LOC158435 | 1.44 | 0.92 | |  | | SLC8A1 | | 0.55 | 1.08 |
| LRIG3 | 1.65 | 0.69 | |  | | SLC9A2 | | 0.61 | 1.20 |
| LRRC4C | 1.65 | 0.94 | |  | | SMIM19 | | 0.75 | 1.10 |
| LSS | 1.38 | 0.89 | |  | | SORBS2 | | 0.53 | 1.17 |
| LTBP2 | 1.64 | 0.82 | |  | | SOX11 | | 0.48 | 1.10 |
| MAP3K2 | 1.06 | 0.87 | |  | | SPATA6L | | 0.64 | 1.03 |
| MAP3K7CL | 2.22 | 0.93 | |  | | SPC24 | | 0.58 | 1.18 |
| MAPKAP1 | 1.98 | 0.92 | |  | | SRGAP3 | | 0.63 | 1.27 |
| MED17 | 1.75 | 0.85 | |  | | ST6GALNAC2 | | 0.72 | 1.22 |
| MIR2113 | 1.53 | 0.86 | |  | | SYK | | 0.59 | 1.11 |
| MIRLET7BHG | 1.94 | 0.94 | |  | | TMCC3 | | 0.76 | 1.07 |
| NBEAL1 | 2.11 | 0.85 | |  | | TRAP1 | | 0.72 | 1.15 |
| NCAPD3 | 2.07 | 0.95 | |  | | TROAP | | 0.91 | 1.15 |
| NGDN | 1.48 | 0.88 | |  | | UBE2D4 | | 0.81 | 1.07 |
| NREP | 1.58 | 0.94 | |  | | UQCR10 | | 0.89 | 1.18 |
| OAT | 1.28 | 0.87 | |  | | WFS1 | | 0.54 | 1.10 |
| OSBPL5 | 1.44 | 0.92 | |  | | YDJC | | 0.73 | 1.11 |
| OSBPL8 | 1.75 | 0.83 | |  | | ZBTB33 | | 0.51 | 1.19 |
| PAPD7 | 1.76 | 0.93 | |  | | ZNF185 | | 0.60 | 1.21 |
| PAWR | 1.31 | 0.89 | |  | | ZNF677 | | 0.71 | 1.06 |
| PDIA6 | 1.53 | 0.85 | |  | |  | |  |  |
| PGM3 | 1.52 | 0.73 | |  | |  | |  |  |
| PLCH2 | 1.62 | 0.86 | |  | |  | |  |  |
| PMFBP1 | 1.34 | 0.91 | |  | |  | |  |  |
| PNRC1 | 1.77 | 0.92 | |  | |  | |  |  |
| PPP3CC | 1.33 | 0.93 | |  | |  | |  |  |
| PRH1 | 1.92 | 0.87 | |  | |  | |  |  |
| PSMB4 | 1.41 | 0.95 | |  | |  | |  |  |
| PTPN3 | 2.06 | 0.88 | |  | |  | |  |  |
| PURB | 2.02 | 0.81 | |  | |  | |  |  |
| PXK | 1.17 | 0.85 | |  | |  | |  |  |
| RAB2A | 1.82 | 0.89 | |  | |  | |  |  |
| RECK | 1.28 | 0.87 | |  | |  | |  |  |
| RECQL | 1.78 | 0.84 | |  | |  | |  |  |
| SDK1 | 1.39 | 0.94 | |  | |  | |  |  |
| SEC16A | 3.40 | 0.78 | |  | |  | |  |  |
| SFXN1 | 1.53 | 0.92 | |  | |  | |  |  |
| SLC13A5 | 1.30 | 0.83 | |  | |  | |  |  |
| SLC1A5 | 2.21 | 0.90 | |  | |  | |  |  |
| SMG9 | 2.58 | 0.95 | |  | |  | |  |  |
| SNRPN | 2.97 | 0.89 | |  | |  | |  |  |
| SNX29 | 1.59 | 0.90 | |  | |  | |  |  |
| SPECC1 | 1.33 | 0.93 | |  | |  | |  |  |
| ST7 | 1.59 | 0.92 | |  | |  | |  |  |
| SUGP2 | 1.44 | 0.95 | |  | |  | |  |  |
| SURF4 | 1.28 | 0.87 | |  | |  | |  |  |
| TASP1 | 1.68 | 0.90 | |  | |  | |  |  |
| TBC1D19 | 1.98 | 0.86 | |  | |  | |  |  |
| TENM3 | 1.07 | 0.83 | |  | |  | |  |  |
| TEX2 | 1.37 | 0.84 | |  | |  | |  |  |
| TGFB1I1 | 1.45 | 0.86 | |  | |  | |  |  |
| TMCC1 | 1.48 | 0.71 | |  | |  | |  |  |
| TMEM117 | 2.49 | 0.84 | |  | |  | |  |  |
| TNFAIP3 | 1.91 | 0.74 | |  | |  | |  |  |
| TNFAIP6 | 1.54 | 0.95 | |  | |  | |  |  |
| TNFRSF10D | 1.89 | 0.94 | |  | |  | |  |  |
| TNKS1BP1 | 1.96 | 0.94 | |  | |  | |  |  |
| TTC28 | 1.37 | 0.93 | |  | |  | |  |  |
| URI1 | 1.39 | 0.91 | |  | |  | |  |  |
| VASN | 1.33 | 0.87 | |  | |  | |  |  |
| WIPI1 | 1.44 | 0.78 | |  | |  | |  |  |
| ZBED6 | 1.69 | 0.89 | |  | |  | |  |  |
| ZBTB9 | 1.24 | 0.94 | |  | |  | |  |  |
| ZFP30 | 1.65 | 0.91 | |  | |  | |  |  |
| ZNF331 | 1.75 | 0.96 | |  | |  | |  |  |

*****FC, Fold change.

**Table S2: Common hypermethylation CGs containing CTCF binding sites in both prostate and breast tumors**

|  |  | **Prostate DM CGs in CTCF Sites** | | | |  |  | **Breast DM CGs in CTCF Sites** | | | |
| --- | --- | --- | --- | --- | --- | --- | --- | --- | --- | --- | --- |
| **Chr** | **Gene_Symbol** | **LNCaP_start** | **LNCaP_stop** | **LNCaP_id** | **CG_Name** | **Genomic**  **Coordinate** |  | **MCF_start** | **MCF_stop** | **MCF_id** | **CG_Name** |
| **1** |  | 2246644 | 2246885 | chr1.124 | cg10195763 | 2246864 | <- Overlap -> | 2246685 | 2246875 | MCF91 | cg10195763 |
| **1** |  | 85773844 | 85774174 | chr1.2464 | cg11348338 | 85774063 | <- Overlap -> | 85773915 | 85774112 | MCF1988 | cg11348338 |
| **11** | PLEKHA7 | 16809965 | 16810243 | chr11.546 | cg07328635 | 16810103 | <- Overlap -> | 16810014 | 16810200 | MCF8149 | cg07328635 |
| **19** | AP1M2 | 10691664 | 10691995 | chr19.533 | cg10516832 | 10691895 | <- Overlap -> | 10691737 | 10691937 | MCF25955 | cg10516832 |
| **19** | AP1M2 | 10691664 | 10691995 | chr19.533 | cg23652172 | 10691765 | <- Overlap -> | 10691737 | 10691937 | MCF25955 | cg10516832 |
| **19** | AP1M2 | 10691664 | 10691995 | chr19.533 | cg10516832 | 10691895 | <- Overlap -> | 10691737 | 10691937 | MCF25955 | cg26999505 |
| **19** | AP1M2 | 10691664 | 10691995 | chr19.533 | cg23652172 | 10691765 | <- Overlap -> | 10691737 | 10691937 | MCF25955 | cg26999505 |
| **19** | AP1M2 | 10691664 | 10691995 | chr19.533 | cg10516832 | 10691895 | <- Overlap -> | 10691737 | 10691937 | MCF25955 | cg14985481 |
| **19** | AP1M2 | 10691664 | 10691995 | chr19.533 | cg23652172 | 10691765 | <- Overlap -> | 10691737 | 10691937 | MCF25955 | cg14985481 |
| **2** |  | 20335434 | 20335668 | chr2.419 | cg07142377 | 20335552 | <- Overlap -> | 20335468 | 20335658 | MCF28280 | cg07142377 |
| **2** | NIF3L1 | 201766243 | 201766540 | chr2.3840 | cg09917123 | 201766393 | <- Overlap -> | 201766254 | 201766444 | MCF31394 | cg09917123 |
| **2** | AGAP1 | 236504127 | 236504386 | chr2.4576 | cg03742137 | 236504280 | <- Overlap -> | 236504192 | 236504317 | MCF32107 | cg03742137 |
| **20** | ATP9A | 50255817 | 50256110 | chr20.1328 | cg26048101 | 50255874 | <- Overlap -> | 50255867 | 50256072 | MCF33995 | cg26048101 |
| **22** | ARVCF | 19958031 | 19958303 | chr22.139 | cg20291779 | 19958201 | <- Overlap -> | 19958081 | 19958268 | MCF35647 | cg20291779 |
| **7** |  | 50485775 | 50486115 | chr7.1204 | cg25823373 | 50485923 | <- Overlap -> | 50485840 | 50486049 | MCF48808 | cg25823373 |
| **11** |  | 126873250 | 126873415 | chr11.3169 | cg00373707 | 126873379 | <- Overlap -> | 12687310 | 126873700 | MCF10414 | cg15436551 |

**Table S3: Differentially methylated CGs in both prostate and breast tumors**

|  |  | **Prostate DM CGs** | | | | |  | **Breast DM CGs** | | | | |
| --- | --- | --- | --- | --- | --- | --- | --- | --- | --- | --- | --- | --- |
| **Chr** | **Gene_Symbol** | **LNCaP_start** | **LNCaP_stop** | **LNCaP_id** | **CG_Name** | **CTCF binding_dist** | **Genomic_**  **Coordinate** | **MCF_start** | **MCF_stop** | **MCF_id** | **CG_Name** | **CTCF binding_dist** |
| **10** | LOC731789 | 26934923 | 26934938 | chr10.563 | cg00241002 | 3011 | 26931912 | 26936644 | 26936834 | MCF5420 | cg00241002 | 4732 |
| **11** |  | 126873250 | 126873415 | chr11.3169 | cg00373707 | 0 | 126873379 | 126873510 | 126873700 | MCF10414 | cg00373707 | 131 |
| **12** | CCDC63 | 111284881 | 111285097 | chr12.2463 | cg01805574 | 81 | 111284800 | 111284898 | 111285003 | MCF12590 | cg01805574 | 98 |
| **8** | PLEC1 | 145049032 | 145049318 | chr8.2972 | cg02110858 | 20810 | 145028222 | 145027868 | 145028058 | MCF53428 | cg02110858 | -164 |
| **2** |  | 170646389 | 170646750 | chr2.3276 | cg02928644 | 0 | 170646566 | 170646578 | 170646677 | MCF31003 | cg02928644 | 12 |
| **2** | AGAP1 | 236504127 | 236504386 | chr2.4576 | cg03742137 | 0 | 236504280 | 236504192 | 236504317 | MCF32107 | cg03742137 | 0 |
| **17** | C17orf50 | 34091791 | 34091806 | chr17.1065 | cg05173913 | -95 | 34091901 | 34091694 | 34091884 | MCF22465 | cg05173913 | -17 |
| **3** | MLH1;EPM2AIP1 | 37034314 | 37034474 | chr3.708 | cg05845319 | 248 | 37034066 | 37034322 | 37034512 | MCF37507 | cg05845319 | 256 |
| **16** | ZNF598 | 2053837 | 2054163 | chr16.128 | cg07078095 | 0 | 2054141 | 2053875 | 2054116 | MCF19018 | cg07078095 | -25 |
| **2** |  | 20335434 | 20335668 | chr2.419 | cg07142377 | 0 | 20335552 | 20335468 | 20335658 | MCF28280 | cg07142377 | 0 |
| **2** | SEMA4C | 97534716 | 97534993 | chr2.1854 | cg07166409 | 659 | 97534057 | 97534293 | 97534483 | MCF29776 | cg07166409 | 236 |
| **11** | PLEKHA7 | 16809965 | 16810243 | chr11.546 | cg07328635 | 0 | 16810103 | 16810014 | 16810200 | MCF8149 | cg07328635 | 0 |
| **6** |  | 108434711 | 108434957 | chr6.2235 | cg08624680 | -13 | 108434970 | 108434787 | 108434977 | MCF46807 | cg08624680 | 0 |
| **15** |  | 89943223 | 89943261 | chr15.1994 | cg08631504 | -170 | 89943431 | 89943169 | 89943298 | MCF18436 | cg08631504 | -133 |
| **6** | PRSS35 | 84224668 | 84224904 | chr6.1888 | cg08687825 | 2916 | 84221752 | 84221633 | 84221825 | MCF46570 | cg08687825 | 0 |
| **5** |  | 133747521 | 133747817 | chr5.2284 | cg09101941 | -27441 | 133775258 | 133775095 | 133775285 | MCF43805 | cg09101941 | 0 |
| **11** | ME3 | 86387622 | 86387832 | chr11.2264 | cg09193479 | 4722 | 86382900 | 86382786 | 86382976 | MCF9803 | cg09193479 | 0 |
| **2** | NIF3L1 | 201766243 | 201766540 | chr2.3840 | cg09917123 | 0 | 201766393 | 201766254 | 201766444 | MCF31394 | cg09917123 | 0 |
| **1** |  | 2246644 | 2246885 | chr1.124 | cg10195763 | 0 | 2246864 | 2246685 | 2246875 | MCF91 | cg10195763 | 0 |
| **19** | AP1M2 | 10691664 | 10691995 | chr19.533 | cg10516832 | 0 | 10691895 | 10691737 | 10691937 | MCF25955 | cg10516832 | 0 |
| **8** | TNFRSF10D | 23020954 | 23021198 | chr8.610 | cg10964421 | 0 | 23021093 | 23082458 | 23082648 | MCF51242 | cg10964421 | 61365 |
| **14** | BCL11B | 99738517 | 99738745 | chr14.1773 | cg11065634 | 1539 | 99736978 | 99738565 | 99738755 | MCF16146 | cg11065634 | 1587 |
| **1** |  | 85773844 | 85774174 | chr1.2464 | cg11348338 | 0 | 85774063 | 85773915 | 85774112 | MCF1988 | cg11348338 | 0 |
| **15** | UBE2QP1 | 85114067 | 85114279 | chr15.1860 | cg11777390 | 477 | 85113590 | 85114290 | 85114447 | MCF18287 | cg11777390 | 700 |
| **3** | USP4 | 49378112 | 49378223 | chr3.1088 | cg12384572 | 161 | 49377951 | 49378069 | 49378259 | MCF37822 | cg12384572 | 118 |
| **1** | RGS2 | 192776825 | 192776848 | chr1.4507 | cg18150280 | -11 | 192776859 | 192777711 | 192777901 | MCF3711 | cg18150280 | 852 |
| **1** | FAM63A | 150979292 | 150979471 | chr1.3427 | cg18182216 | 907 | 150978385 | 150978893 | 150979083 | MCF2709 | cg18182216 | 508 |
| **5** | ARL15 | 53607736 | 53607898 | chr5.896 | cg18784506 | 125 | 53607611 | 53607721 | 53607911 | MCF42956 | cg18784506 | 110 |
| **17** |  | 25758005 | 25758249 | chr17.787 | cg18918921 | -24722 | 25782971 | 25782945 | 25783135 | MCF22224 | cg18918921 | 0 |
| **12** | CCDC63 | 111284881 | 111285097 | chr12.2463 | cg19006003 | 55 | 111284826 | 111284898 | 111285003 | MCF12590 | cg19006003 | 72 |
| **15** | ARID3B | 74836014 | 74836176 | chr15.1498 | cg19144497 | 0 | 74836094 | 74794757 | 74794947 | MCF17898 | cg19144497 | -41147 |
| **22** | ARVCF | 19958031 | 19958303 | chr22.139 | cg20291779 | 0 | 19958201 | 19958081 | 19958268 | MCF35647 | cg20291779 | 0 |
| **3** | MLH1;EPM2AIP1 | 37034314 | 37034474 | chr3.708 | cg21109167 | 230 | 37034084 | 37034322 | 37034512 | MCF37507 | cg21109167 | 238 |
| **6** | HMGCLL1 | 55408886 | 55408980 | chr6.1473 | cg21705897 | -35081 | 55444061 | 55443851 | 55444041 | MCF46376 | cg21705897 | -20 |
| **17** | RND2 | 41158665 | 41158820 | chr17.1362 | cg22325646 | -17649 | 41176469 | 41176530 | 41176627 | MCF22724 | cg22325646 | 61 |
| **12** | ASB8 | 48551310 | 48551539 | chr12.1074 | cg22385764 | -55 | 48551594 | 48551381 | 48551478 | MCF11303 | cg22385764 | -116 |
| **8** | TNFRSF10D | 23021252 | 23021294 | chr8.611 | cg22783363 | -259 | 23021553 | 23082458 | 23082648 | MCF51242 | cg22783363 | 60905 |
| **8** | EBF2 | 25867730 | 25867937 | chr8.683 | cg22987487 | -139 | 25868076 | 25867751 | 25867941 | MCF51286 | cg22987487 | -135 |
| **2** | PTH2R | 209272500 | 209272733 | chr2.4001 | cg23524735 | 1003 | 209271497 | 209272505 | 209272695 | MCF31534 | cg23524735 | 1008 |
| **6** | RBM24 | 17281459 | 17281673 | chr6.430 | cg25302957 | -611 | 17282284 | 17281516 | 17281615 | MCF45369 | cg25302957 | -669 |
| **9** | GALT | 34646855 | 34647168 | chr9.612 | cg25476145 | 0 | 34646939 | 34646957 | 34647082 | MCF53909 | cg25476145 | 18 |
| **17** | RND2 | 41158665 | 41158820 | chr17.1362 | cg25504443 | -17653 | 41176473 | 41176530 | 41176627 | MCF22724 | cg25504443 | 57 |
| **7** |  | 50485775 | 50486115 | chr7.1204 | cg25823373 | 0 | 50485923 | 50485840 | 50486049 | MCF48808 | cg25823373 | 0 |
| **1** | PTGS2 | 186649461 | 186649519 | chr1.4439 | cg25837803 | -390 | 186649909 | 186649401 | 186649540 | MCF3678 | cg25837803 | -369 |
| **20** | ATP9A | 50255817 | 50256110 | chr20.1328 | cg26048101 | 0 | 50255874 | 50255867 | 50256072 | MCF33995 | cg26048101 | 0 |
| **17** | MAPT | 44050772 | 44050860 | chr17.1500 | cg26163368 | 24120 | 44026652 | 44026516 | 44026706 | MCF22864 | cg26163368 | 0 |
| **1** | JAK1 | 65327394 | 65327485 | chr1.2125 | cg26315985 | -36385 | 65363870 | 65360620 | 65360810 | MCF1864 | cg26315985 | -3060 |
| **6** |  | 130686481 | 130686737 | chr6.2652 | cg26483578 | 0 | 130686708 | 130618117 | 130618307 | MCF47054 | cg26483578 | -68401 |

**Table S4: Array Validation of Cytoscan Probes**

| **Cytoscan Probe** | ***NspI* Fragment**  **Chr:Start-Stop** | **Methylation Fold Change** | ***P-*value** | **CpG Percentage** |
| --- | --- | --- | --- | --- |
| **C-4QPFF** | Chr8:67782483-67782995 | 1.85 | 0.01 | 4.29 |
| **C-6LWFW** | Chr5:141184952-141185538 | 2.34 | 2.7E-04 | 1.02 |
| **C-4QXQN** | Chr7:28893607-28894301 | 1.71 | 1.7E-03 | 6.62 |
| **C-3GEQV** | Chr4:118569881-118570276 | 2.08 | 1.12E-05 | 1.01 |
| **C-6XOOB** | Chr6:12289044-12289931 | 1.71 | 0.016 | 1.24 |

**Table S5: Combined Bisulfite Restriction Analysis (COBRA) Assay Characteristics (Related to Additional file 1: Fig. S1)**

| **Cytoscan Probe** | **Bisulfite Converted Sequence** | **Amplicon Size;**  **Enzyme**  **Cut Site(s)** |
| --- | --- | --- |
| **C-4QPFF** | **5’-GGGAATATTGGAATGTTTTTTA**AAGTGATTAGATATTTTGTATTTTTTGAAGTAGCGTTTTGAATTTTTTAAATATAATATTATTTGTTTGAGATAATAATAAAAATTAAACGTAGATTTATTTTTTTTTTGTT**TCGA**TGGGAGGGAGTAAGAGTGAAGTTATTAAAACGTAAGGGAATAAGTTCGGTTAGTTTTTTTGTTGAT**TCGA**GGGCGCGGGCGGTTTCGGTAGGTTTCGCGACGTAGTTAACGGTCGGGACGTGCGCGTATGCGCGTTAGGATTTCGTTTCGTTTACGTTGTAGGCGGAGAGTAATC**GTTAAGTTTGGTGGGAGTTAAG**  **-3’** | 335bp;  *TaqI*;  135, 205 |
| **C-6LWFW** | **5’-ATTTTTGGTTGTATTTGAGTAAAA**TTTTAGGAAGGGACGTATAGTTTGATTGTGTTGGAAGGAAGGTAGAAGGATGGATAGGGAGATTTTGAAGATGTTTTCGTAGGATTAGAGAAGGAGGTAGCGGAGGTATAGTAGTTTTTTT**TCGA**GGTTTTTTTTGAGATTTATTTTTTGGAGGTTTTGTCGGAATTTACGGTGATT**TTGAAGTAGGGGTTATGTTTT**  **-3’** | 222bp;  *TaqI*;  146 |
| **C-4QXQN** | **5’-**  **GAAGGAGGTAGTTTTGGTAGTT**AGGGAAGGTGCGGTGTTCGTGTCGGGGTCGCGGGGCGTGCGGGAGTTTTGGGCGTCGTCGTATTTGTCGGTTTTTTTTGGGAGTTTTGGG**TCGA**ATTTTTAGTTCGGGCGTTTTTTGGCGTCGTCGTGGGGAAATTATTCGGCGTTAGGCGTTATGTCGTATAATATTGTGGAGTAGTTTTGTCGGCGGGGTGGGTGTTTTTTATTTAAGGTATTGGATTAGGGGTTTTGTTAGTAAATATGGTTTTTTTATTAGCGTGTCGGGGT**TGTAGAATTTTAGGGTATGGTTT**  **-3’** | 311bp;  *TaqI*;  113 |
| **C-3GEQV** | **5’-**  **TTGGAGTTTTTTTTTTAGTTTTT**TGTTTTGATGATTCGTTTAATGTTGTTAG**CGCG**TG**TCGA**AGTTTTTTATTATTATTATGTTGTTGTTTAAGTTGTTTTATAGGTTAAGAAGAATTTGTTTTATGATT**TTGGGTATTTTAATGTT**  **-3’** | 147bp;  *BstUI* – 54;  *TaqI* – 59 |
| **C-6XOOB** | **5’-ATTGTTTTGTGTGTATTTTAGG**GGGGGATTTTAAGGTTAGATAGATAGGAAATTGTTTTGAAAATGTAAATATATTATTAAATGTGAAGTATTATTTGATTTTTTGT**TCGA**ATGGTATTTTTTTTTTAGTATTATTTTTTTTGTATATTTATTTAATTTTGTATAAGAATATTTTTTTGTTTTAAATGAAGATATTTTTTTAAAAAAAAGAGTTTTAGAAAATATGTTTTTGTTTGTGCGGGGAATAAATAGAATATTTTGAGGTGTATTTTTTTTTTTTATGTTAGGTAATATTTTTTGATTTTTTTCGGTTTTTAAGTTAGGTTGCGTTT**TTTTTTGTTATTTAGAAGGGTT**  **-3’** | 354bp;  *TaqI*;  108 |

**Table S6: Primers Used for Validation of Cytoscan Array (Related to Additional file 1: Fig. S1)**

| **Cytoscan Probe** | **Method** | **Primer Sequence**  **(5’->3’)** |
| --- | --- | --- |
| **C-4QPFF** | COBRA | For – GGGAATATTGGAATGTTTTTTA  Rev – CTTAACTCCCACCAAACTTAAC |
| **C-4QPFF** | MeDIP-qPCR | For – TTTCTCTGCTTCGCTGGGAG  Rev – CACTCACCTTGACTCCCACC |
| **C-6LWFW** | COBRA | For – ATTTTTGGTTGTATTTGAGTAAAA  Rev – AAAACATAACCCCTACTTCAA |
| **C-6LWFW** | MeDIP-qPCR | For – GCAACAACAGGCCTAGTCCT  Rev – AATAGAGCTGTGGCCCAGTG |
| **C-4QXQN** | COBRA | For – GAAGGAGGTAGTTTTGGTAGTT  Rev – AAACCATACCCTAAAATTCTACA |
| **C-4QXQN** | MeDIP-qPCR | For – CTTGGTAGCCAGGGAAGGTG  Rev – AACCATGCCCTGAGGTTCTG |
| **C-3GEQV** | COBRA | For – TTGGAGTTTTTTTTTTAGTTTTT  Rev – AACATTAAAATACCCAAAATCA |
| **C-3GEQV** | MeDIP-qPCR | For – TCTGCCCTGATGATCCGTCT  Rev – AGAAGGGCAAAGAAGGACATT |
| **C-6XOOB** | COBRA | For – ATTGTTTTGTGTGTATTTTAGG  Rev – AACCCTTCTAAATAACAAAAAA |
| **C-6XOOB** | MeDIP-qPCR | For – TGCCCTAAATGAAGACACCCC  Rev – GGTCGGTGCCACCAATCTTA |

**Table S7: MeDIP-qPCR Performed region for Cytoscan Array Validation and methylation values (Related to Additional file 1: Fig. S1)**

| **Cytoscan Probe** | **MeDIP-qPCR Region** | **shCTCF1 FC;**  ***P*-value** | **shCTCF2 FC;**  ***P*-value** |
| --- | --- | --- | --- |
| **C-4QPFF** | chr8:67782856-67783073 | 2.79 Fold;  3.6E-05 | 3.92 Fold;  7.0E-04 |
| **C-6LWFW** | chr5:141185284-141185572 | 2.37 Fold;  2.5E-05 | 1.44 Fold;  0.023 |
| **C-4QXQN** | chr7:28,893,861-28,894,158 | 2.44 Fold;  6.9E-04 | 1.38 Fold;  4.3E-03 |
| **C-3GEQV** | chr4:118570146-118570346 | 2.05 Fold;  1.0E-04 | 1.56 Fold;  1.4E-03 |
| **C-6XOOB** | chr6:12289526-12289797 | 2.23 Fold;  6.6E-05 | 1.51 Fold;  8.0E-3 |

**Table S8: Primers Used for ChIP-qPCR and MeDIP-qPCR in Extended CTCF Knockdown Studies (Related to Fig. 3 and Additional file 1: Fig. S3)**

| **Gene of pCBS;**  **Genome Location (hg19)** | **Sequence (5’ -> 3’)** | **Amplicon Length** |
| --- | --- | --- |
| LTBP2  Chr14:75079440-75079547 | For – GGAGCGCAGGGAGGTCC  Rev – AGGGGATTGAGATCCAGAGC | 108bp |
| TNFAIP3;  Chr6:138190414-138190862 | For – GCTGTGCCACAAAGGAAAGG  Rev – TGCTGGGAAAGGCATAGTGG | 139bp |
| FGF5;  Chr4:81188069-81188400 | For – TGGAGCAGAGCAGTTTCCAG  Rev – ATTGACTTTGCCATCCGGGT | 110bp |
| EPHA3;  Chr3:89148142-89148548 | For – AAGTTAAGCGAACACGAGCG  Rev – GTCCAGGTGGCAAGGAGTAC | 95bp |
| AMIGO2;  Chr12:47473167-47473554 | For – TCCGTGTCTGTCACTCTTGC  Rev – CGGCTGCTTGAAACTCCTCA | 103bp |
